# Antidiabetic drugs in Parkinson’s disease: a comprehensive meta-analysis on efficacy and safety with trial sequential analysis and GRADE evaluation

**DOI:** 10.1007/s10787-025-01873-0

**Published:** 2025-08-05

**Authors:** Amr M. Abou Elezz, Kareem Khalefa, Ahmed Farid Gadelmawla, Youssef A. Khattab, Mohamed Abo Zeid

**Affiliations:** 1 Faculty of Medicine, Tanta University, Tanta, Egypt; 2 Faculty of Medicine, Menoufia University, Menoufia, Egypt; 3 Faculty of Medicine, Mansoura University, Mansoura, Egypt

**Keywords:** Antidiabetic agent, Parkinson’s disease, Meta-analysis, Glucagon-like peptide, Exenatide, Lixisenatide, Thiazolidinedione

## Abstract

Recent studies highlighted the relation between type 2 diabetes and Parkinson’s disease, suggesting a relation between insulin resistance and *α*-synuclein aggregation. Antidiabetic medications, including GLP-1 receptor agonists and PPAR-*γ* agonists, have shown potential neuroprotective effects. We conducted a comprehensive literature search retrieving randomized controlled trials (RCTs) comparing antidiabetic drugs and placebo. Key outcomes included motor and non-motor symptoms, along with the safety profile. Data were analyzed using RevMan, and trial sequential analysis as well as sensitivity analysis were conducted to ensure the robustness of our results. In addition, to ensure the reliability of our evidence, we conducted the GRADE evaluation approach. Seven RCTs, with 973 patients, were eligible for our inclusion criteria. Antidiabetic drugs have shown no significant difference from placebo concerning change in MDS-UPDRS scores while on medication in Parts I, II, III, IV (MD = −0.04, 95% CI [−0.74 to 0.66], *p* = 0.90), (MD = −0.88, 95% CI [−2.11 to 0.34], *p* = 0.16), (MD = −1.10, 95% CI [−2.61 to 0.42], *p* = 0.16), (MD = −0.09, 95% CI [−0.45 to 0.27], *p* = 0.64), respectively. However, for MATTIS-DRS and MADRS scores, results showed a significant difference favoring GLP-1 agonists (MD = 2.42, 95% CI [0.01 to 4.83], *p* = 0.05), (MD = −2.08, 95% CI [−3.93 to −0.23], *p* = 0.03) respectively. As for safety profile, results revealed significant differences favoring the placebo group. This meta-analysis concludes that antidiabetic drugs in early-to mid-stage Parkinson’s disease show no significant benefit considering non-motor symptoms detected by MDS-UPDRS I, with TSA confirming this finding as a conclusive result. Similarly, no notable effects on motor symptoms were observed, although future trials are needed. GLP-1 agonists revealed potential antidepressant effects as well as improving cognitive functions detected by MADRS and MATTIS-DRS, respectively. However, antidiabetic drugs were associated with higher risks of gastrointestinal adverse effects such as nausea, vomiting, and weight loss.

**Supplementary Information:**

The online version contains supplementary material available at 10.1007/s10787-025-01873-0.

## Introduction

Parkinson’s disease (PD) is a progressive neurodegenerative condition characterized by degeneration of multiple neurotransmitters, particularly the dopaminergic nigrostriatal pathway (Armstrong and Okun 2020). It is thought to be associated with the cumulative effects of toxic *α*-synuclein species, which are thought to misfold, aggregate, and trigger various pathological responses, such as inflammation and microglial activation (Ono 2017; Brás and Outeiro 2021; Ferreira and Romero-Ramos 2018) leading to persistent misfolding, neuronal dysfunction, and ultimately, neurodegeneration (Garcia et al. 2022).

PD affects approximately 1% of the world population above the age of 60 (Lau and Breteler 2006). It shows primary motor symptoms including bradykinesia, rigidity, tremors, and postural instability, leading to gait disturbances. It also shows a range of non-motor symptoms such as cognitive impairment, depression, autonomic dysfunction, and hyposmia further complicating the course of the disease. It is commonly known that all traditional medications aim to replenish dopamine and restore dopaminergic function to alleviate symptoms at different stages; nevertheless, no widely accepted strategies exist to alter the progressive course of the disease (Olanow et al. 2009).

Various epidemiological analyses have suggested that individuals with type 2 diabetes mellitus (DM) have an increased likelihood of developing Parkinson’s disease compared to those without DM (Cullinane et al. 2023). As there is a consensus that inflammation has a pivotal role in this overlapping pathology of type 2 diabetes and Parkinson’s disease, DM has been shown to impair mitochondrial function within microglial cells, causing mitochondrial DNA (mtDNA) to leak into the cytoplasm thus triggering the cGAS–STING signaling pathway, which in turn activates a robust inflammatory response. The resulting pro-inflammatory cytokines released by activated microglia contribute to dopaminergic neuronal injury, accelerating the neurodegenerative processes characteristic of Parkinson’s disease (PD), pointing to the rationale for antidiabetic drugs—particularly GLP-1 receptor agonists and PPAR-*γ* as promising therapeutic options in PD (Zhang et al. 2024).

Glucagon-like peptide-1 (GLP-1) agonists act by stimulating GLP-1 receptors in response to increased glucose levels to enhance insulin secretion, suppress glucagon release, and delay gastric emptying (Lovshin and Drucker 2009). GLP-1 receptors can be found in the pancreas as well as in multiple other organs, especially the brain (Abu-Hamdah et al. 2009; Rowlands et al. 2018). These GLP-1 analogs (exenatide and Lixisenatide) are capable of crossing the blood–brain barrier and modulating various neuronal pathways (Grieco et al. 2019). Activation of GLP-1 receptors in the brain is thought to have anti-inflammatory effects by reducing microglial activation leading to improvements in both motor and non-motor symptoms (Diz-Chaves et al. 2022; Leon et al. 2017).

Pioglitazone, one of the thiazolidinedione classes, decreases insulin resistance as it is a peroxisome proliferator-activated receptor *γ* (PPAR-*γ*) agonist (Moreno et al. 2004; Nicolakakis et al. 2008). The neuroprotective effects of PPAR-*γ* agonists are not yet well defined; they inhibit the activation of microglia and astrocytes and decrease the production of pro-inflammatory cytokines and nitric oxide (Storer et al. 2005).

The aim of this study is to evaluate the efficacy and safety of antidiabetic medications in relieving both motor and non-motor symptoms in patients with Parkinson’s disease.

## Methods

### Study design and registration

We conducted this systematic review and meta-analysis adhering to the PRISMA (Page et al. 2021) (Preferred Reporting Items for Systematic Reviews and Meta-Analyses) guidelines and following the Cochrane Collaboration’s recommendations. The GRADE approach was used to assess the strength of the evidence (Schünemann et al. 2024). The protocol for this study was registered in the International Prospective Register of Systematic Reviews (Prospero) under the registration number: CRD420251002528.

### Search strategy and study selection process

We conducted a systematic search through the following medical electronic databases: PubMed, Scopus, Web of Science, and Cochrane library including studies up to 11th February 2025 using Medical Subject Headings (MeSH). The search strategy for each database can be found in Table (S1). Duplicates were removed using endnote software; then two independent reviewers (MA and KK) screened the titles and abstracts of the potential studies using Rayyan website (Ouzzani et al. 2016), and the remaining articles were screened based on their full text. Any conflicts were solved by discussion.

### Eligibility criteria

To establish our inclusion criteria, we adopted the PICO framework. We included randomized controlled trials (RCTs) that compared any antidiabetic drug with a placebo. All doses were eligible. We included studies whose patients were diagnosed with Parkinson’s disease, with early-to-mid-stage progression based on the Hoehn & Yahr scale with stages ranging from 1 to 3 based on established clinical criteria. We included studies that reported at least one of the following outcomes: Movement Disorder Society–Unified Parkinson’s Disease Rating Scale (MDS-UPDRS) parts I to IV, Non-Motor Symptoms Scale (NMSS), Parkinson’s Disease Questionnaire-39 (PDQ-39), Mattis Dementia Rating Scale (MATTIS-DRS), Montgomery–Åsberg Depression Rating Scale (MADRS), or any safety outcomes.

We excluded non-randomized control trials, RCTs with irrelevant outcomes, or unavailable complete data, studies that included animal experiments, conference reports, reviews, retrospective studies, meta-analyses, and case reports.

### Quality assessment

We used the Cochrane risk-of-bias tool for randomized trials (RoB-2) (Sterne et al. 2019) to assess the methodological quality of the RCTs. The tool evaluates six key aspects: (1) bias in random sequence generation, (2) bias due to deviations from intended interventions, (3) bias due to missing outcome data, (4) bias in outcome measurement, (5) bias in the selection of reported results, and (6) overall bias. Each aspect is classified as “low risk,” “some concern,” or “high risk.” Two authors independently conducted the risk-of-bias assessment with a third author resolving any disagreements.

### Data extraction

All relevant data were extracted, divided into three main sections: (1) study characteristics, (2) population demographics, and (3) outcome measurements. Data from the included studies were extracted by two authors independently into an online data extraction sheet. A third author was involved in resolving any conflicts.

### Data synthesis and statistical analysis

RevMan Cochrane software (Cumpston et al. 2019), version 5.4, was used to analyze the data. In the absence of heterogeneity, a fixed effect model will be used; otherwise, a random effect model will be used. We analyzed dichotomous data using risk ratio (RR), while continuous data were analyzed using mean difference (MD) with a 95% confidence interval (CI) and *p*-values <0.05 considered statistically significant.

### Assessment of heterogeneity

The Chi-square test was used to assess the presence of significant heterogeneity, with a *p* value of ≤0.1 considered statistically significant heterogeneity. In addition, the *I*^2^ test was used to evaluate heterogeneity according to *I*^2^ classification. *I*^2^ of (≤30%) classified as non-significant heterogeneity, (30–50%) classified as moderate heterogeneity, and (70%) classified as significant heterogeneity.

### Trial sequential analysis

In order to ensure the validity and conclusiveness of our meta-analytic results and minimize the risk of false positive (Type I error), we conducted a trial sequential analysis (TSA) using the Copen-Hagen TSA program version 0.9.5.10 Beta (Thorlund et al. 2017). We set parameters including a type 1 error (*α*) of 5% and type 2 error (*β*) of 20% corresponding to a statistical power of 80%. We used a model variance-based heterogeneity correction.

For each of our primary outcomes (MDS-UPDRS), we generated both a monitoring boundaries plot and a penalized *Z*-curve plot, ensuring the robustness of our conclusions. As for the monitoring boundaries plot, it employed a superiority boundary based on the O’Brien–Fleming alpha spending function to mitigate type 1 errors and a futility boundary based on the O’Brien–Fleming beta spending function to mitigate type 2 errors. Regarding the penalized *Z*-curve plot, the cumulative *z* statistic was penalized by applying the Law of Iterated Logarithm incorporating a *λ* value of 2 (Thorlund et al. 2017). Both plots included a required information size (RIS) axis to determine whether the data are sufficient or not. Data from a low risk-of-bias study were used.

Given the moderate to considerable heterogeneity observed across our primary outcomes (ranging from 50 to 80%) in addition to the low number of included studies, we employed the Biggerstaff–Tweedie (BT) random‐effects model in our TSA. To form the monitoring boundaries, we utilized data from the study Vijiaratnam et al. due to its low risk of bias and sufficient data. Furthermore, we implemented a sensitivity analysis including empirical pooled data from all studies.

Regarding TSA interpretation, accumulative *Z*-curves passing the superiority boundary were interrupted as conclusive true-positive results. As for those crossing the futility boundaries, they were interpreted as conclusive true-negative results. Meanwhile, *Z*-curves only passing the conventional boundaries (*Z* = ±1.96, *p* = 0.05) were interpreted as inconclusive false positives, guided by the RIS axis to determine if further trials are needed.

## Results

### Search results and study selection

**Fig. 1:**
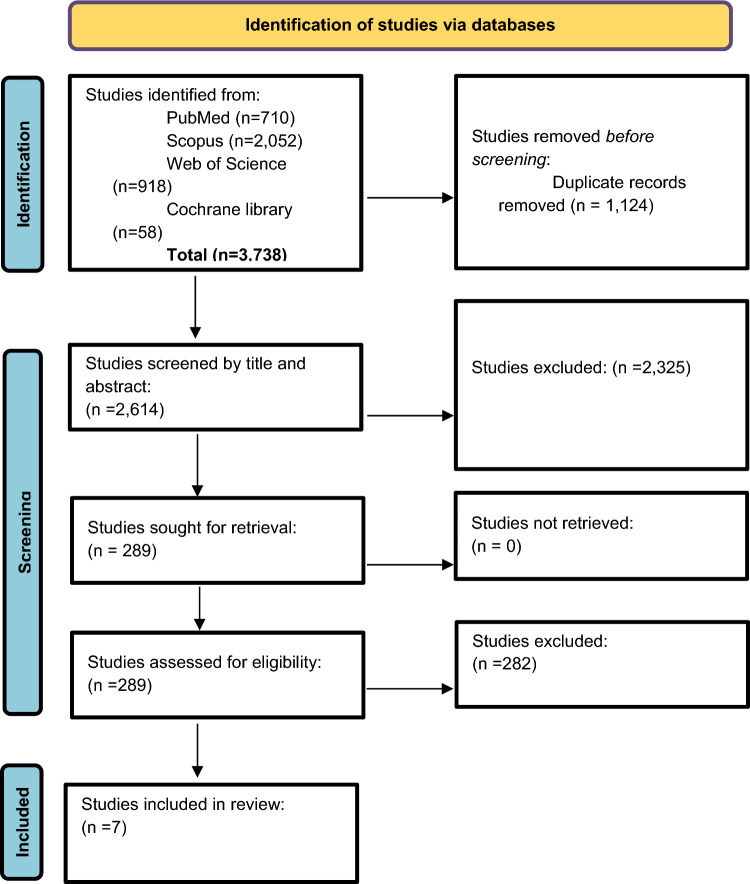
PRISMA flowchart for study selection

Our systematic search yielded 3738 from PubMed, Scopus, WOS, and Cochrane library. After removing 1124 duplicated articles, the remaining 2614 studies went through title and abstract screening. When applying the predefined eligibility criteria, 2325 studies were excluded. The full text of the remaining 289 studies was screened. Eventually, seven studies were included in our study that met our eligibility criteria. The screening process is illustrated in the PRISMA flow diagram (Fig. 1).

### Characteristics of the included studies

Seven RCTs were included in our meta-analysis with 973 patients (433 received GLP-1 agonists, 139 received Thiazolidinedione, 377 received placebo, while the remaining 24 received conventional treatment). Five studies used SC GLP-1 agonists, one used SC GLP-1 agonists, and one used oral Thiazolidinedione) (Table 1). In the intervention group, only 203 (35.5%) were female participants; the mean age was ranging from 63.5 ± 9.8 to 58.8 ± 9.2 years, while the mean duration of PD was ranging from 9.6 ± 3.4 to 0.7 ± 0.7 years. On the other hand, in the control group, only 129 (32.2%) were female participants; the mean age was ranging from 64.2 ± 6.4 to 57.8 ± 8 years, while the mean duration of PD was ranging from 11 ± 5.9 to 0.8 ± 0.7 years (Table 2).

**Table 1:** Summary of the included studies

Study ID	Study design	Antidiabetic drug class	Study arms			Total participants	Country
			Intervention		Control		
			Drug	Route			
McGarry 2024	RCT	GLP-1 Agonist	NLY01 (exenatide analogue) 2.5 mg NLY01 (exenatide analogue) 5 mg	Subcutaneous injections	Placebo	255	United States and Canada
Olmos 2013	RCT	GLP-1 Agonist	Exenatide 20 μg	Subcutaneous injections	Control (conventional PD treatment)	45	United Kingdom
Athauda 2017	RCT	GLP-1 Agonist	Exenatide 2 mg	Subcutaneous injections	Placebo	62	London, United Kingdom
Meissner 2024	RCT	GLP-1 Agonist	Lixisenatide 20 μg	Subcutaneous injections	Placebo	156	France
Hogg. Preprinted 2022	RCT	GLP-1 Agonist	Liraglutide 1.8 mg	Subcutaneous injections	Placebo	63	United States
NET-PD 2015	RCT	Thiazolidinedione	Pioglitazone 15 mg Pioglitazone 45 mg	Oral	Placebo	210	NY, United States
Vijiaratnam 2025	RCT	GLP-1 Agonist	Bydureon (Exenatide analogue) 2 mg	Subcutaneous injections	Placebo	194	London, United Kingdom

*GLP* Glucagon-like peptide-1, *RCT* Randomized controlled trial

**Table 2:** Demographic and baseline characteristics of patients in the included studies

Study ID	Study arms	Study participants	Age Mean (SD)	Sex (female) *n* (%)	Duration of Parkinson’s disease (years)	Hoehn and Yahr stage *n* (%)			MDS-UPDRS score Mean (SD)				
						1	2	2.5	Part I	Part II	Part III	Part IV	Part III off
McGarry (2024)	NLY01 (2.5 mg)	85	62·1 (9·0)	25 (29%)	1.02 (1.04)	22 (26%)	57 (67%)	3 (4%)	4·2 (3·1)	4·8 (3·6)	22·7 (8·1)	N/A	N/A
	NLY01 (5 mg)	85	60·6 (10·0)	31 (36%)	0.96 (0.94)	15 (18%)	60 (71%)	3 (4%)	4·0 (3·7)	5·0 (4·1)	22·0 (8·2)	N/A	N/A
	Placebo	84	61·8 (8·1)	32 (38%)	0.9 (0.98)	14 (17%)	58 (69%)	4 (5%)	4·7 (4·2)	4·9 (3·6)	22·3 (9·1)	N/A	N/A
Olmos (2013)	Exenatide	20	61.4 (6.0)	5 (25%)	9.6 (3.4)	0 (0%)	14 (70%)	6 (30%)	10.4 (4.1)	10.2 (5.2)	31.0 (11.2)	6.3 (2.4)	31.0 (11.2)
	Control	24	59.4 (8.4)	4 (16.7%)	11.0 (5.9)	0 (0%)	16 (66.7%)	8 (33.3%)	11.6 (4.7)	12.9 (6.2)	34.0 (16.1)	6.3 (3.4)	34.0 (16.1)
Athauda (2017)	Exenatide	31	61·6 (8·2)	9 (29%)	6·4 (3·3)	29 (94%)		2 (6%)	9·8 (4·8)	12·5 (6·7)	19·4 (8·4)	4·7 (3·1)	32·8 (9·7)
	Placebo	29	57·8 (8·0)	7 (24%)	6·4 (3·3)	29 (100%)		0 (0%)	9·2 (3·8)	10·7 (5·3)	14·4 (8·2)	5·3 (3·0)	27·1 (10·3)
Meissner (2024)	Lixisenatide	78	59.5 (8.1)	34 (43.58%)	1.4 (0.8)	N/A	N/A	N/A	6.1 (4.0)	5.0 (3.5)	14.8 (7.3)	0.3 (1.3)	N/A
	Placebo	78	59.9 (8.4)	30 (38.46%)	1.4 (0.7)	N/A	N/A	N/A	6.4 (4.2)	5.4 (4.3)	15.5 (7.8)	0.2 (0.8)	N/A
Hogg- Preprinted 2022	Liraglutide	37	63.5 (9.8)	12 (32.4%)	4.7 (3.1)	N/A	N/A	N/A	7.9 (4.8)	8.8 (5.4)	14.8 (7.1)	3.8 (3.3)	26.1 (9.6)
	Placebo	18	64.2 (6.4)	5 (27.8%)	4.8 (3.3)	N/A	N/A	N/A	6.4 (4.1)	7.6 (5.0)	16.3 (9.2)	3.6 (3.2)	28.8 (10.7)
NET-PD (2015)	Pioglitazone (15 mg)	72	61.3 (10.6)	19 (26%)	0.8 (07)	72 (100%)		0	0.8 (0.9)	5.9 (3.2)	17.1 (7.7)	N/A	N/A
	Pioglitazone (45 mg)	67	58.8 (9.2)	20 (30%)	0.7 (0.7)	67 (100%)		0	0.8 (0.9)	5.5 (2.9)	15.0 (7.1)	N/A	N/A
	Placebo	71	59.0 (9.9)	23 (32%)	0.8 (0.7)	71 (100%)		0	0.9 (1.1)	5.5 (3.0)	15.3 (6.5)	N/A	N/A
Vijiaratnam (2025)	Exenatide	97	61·02 (9·05)	28 (29%)	N/A	83 (86%)		14 (14%)	7·9 (4·9)	7·4 (4·9)	20·0 (10·2)	3·9 (3·3)	32·2 (12·5)
	Placebo	97	60·35 (9·26)	28 (29%)	N/A	82 (85%)		15 (15%)	7·5 (4·8)	7·5 (4·9)	20·8 (10·4)	3·8 (3·2)	32·3 (13·3)

*N/A* Not applicable, *MDS-UPDRS* Movement disorder society—unified Parkinson’s disease rating scale, *SD* Standard deviation

### Risk of bias assessment

**Fig. 2:**
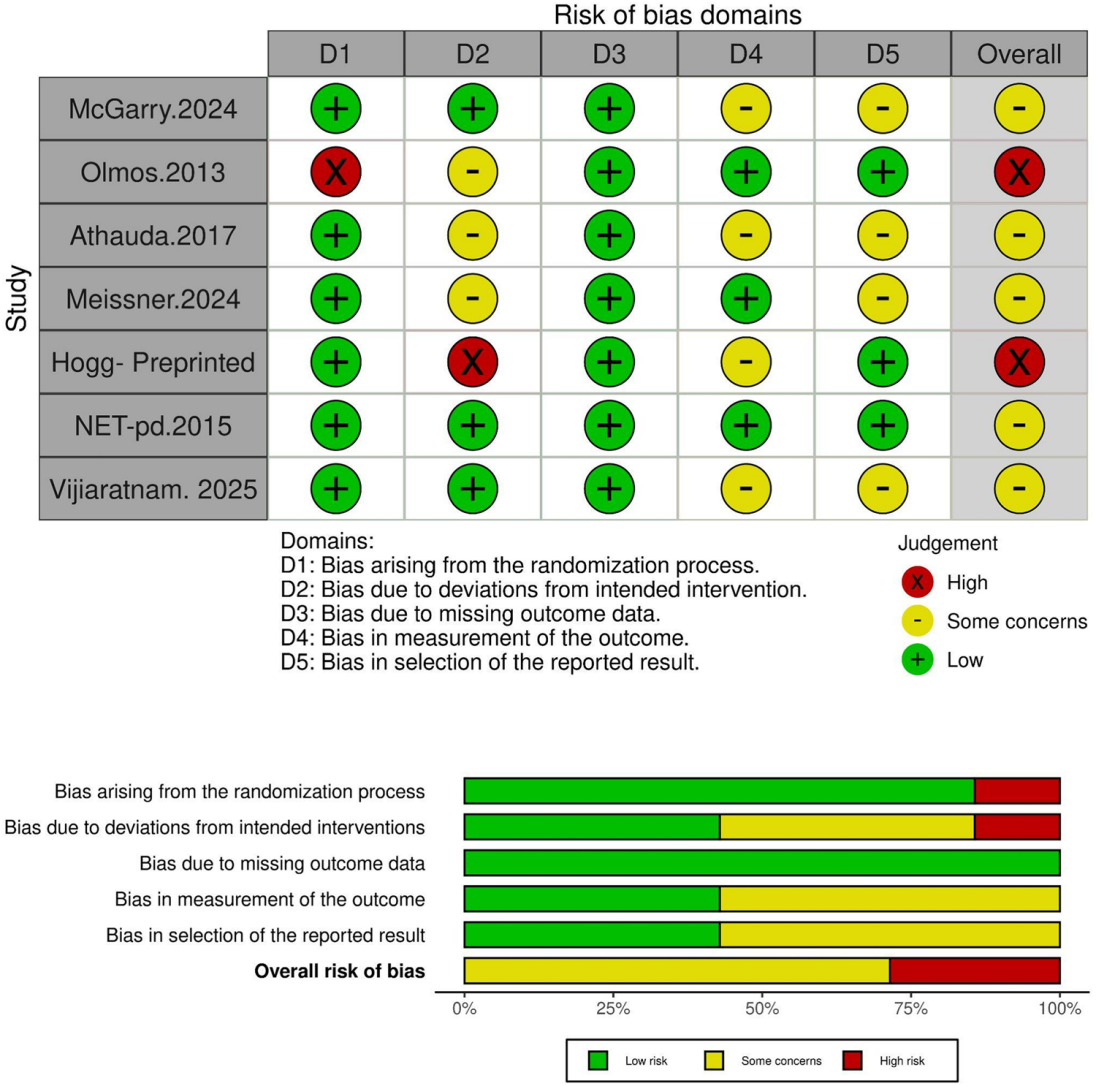
The bias-risk assessment diagram of the included articles

We used Cochrane ROB 2 to evaluate the quality of the included studies, and we found that two studies showed a high risk of bias mainly in the randomization and deviation from intended interventions domains. The remaining five studies showed some concerns (Fig. 2).

### Efficacy outcomes

#### Change from baseline in MDS-UPDRS

We analyzed the four parts of MDS-UPDRS across the studies while patients were on medications between 36 and 54 weeks. In addition, part III (Motor Examination) was specifically analyzed while patients were both on and off medications.

**Fig. 3:**
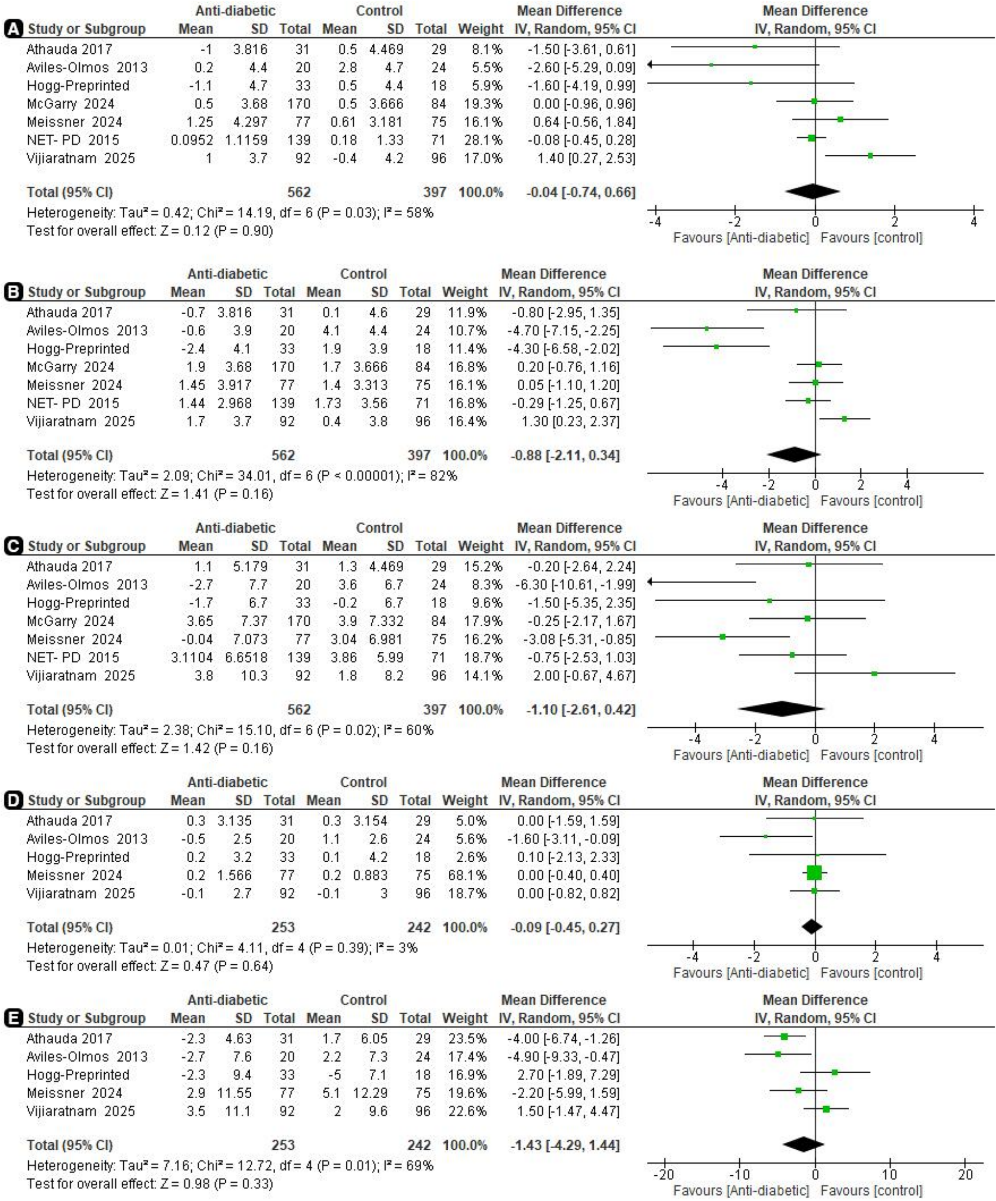
Forest plots comparing (MD) for change from baseline in MDS-UPDRS

**Fig. 4:**
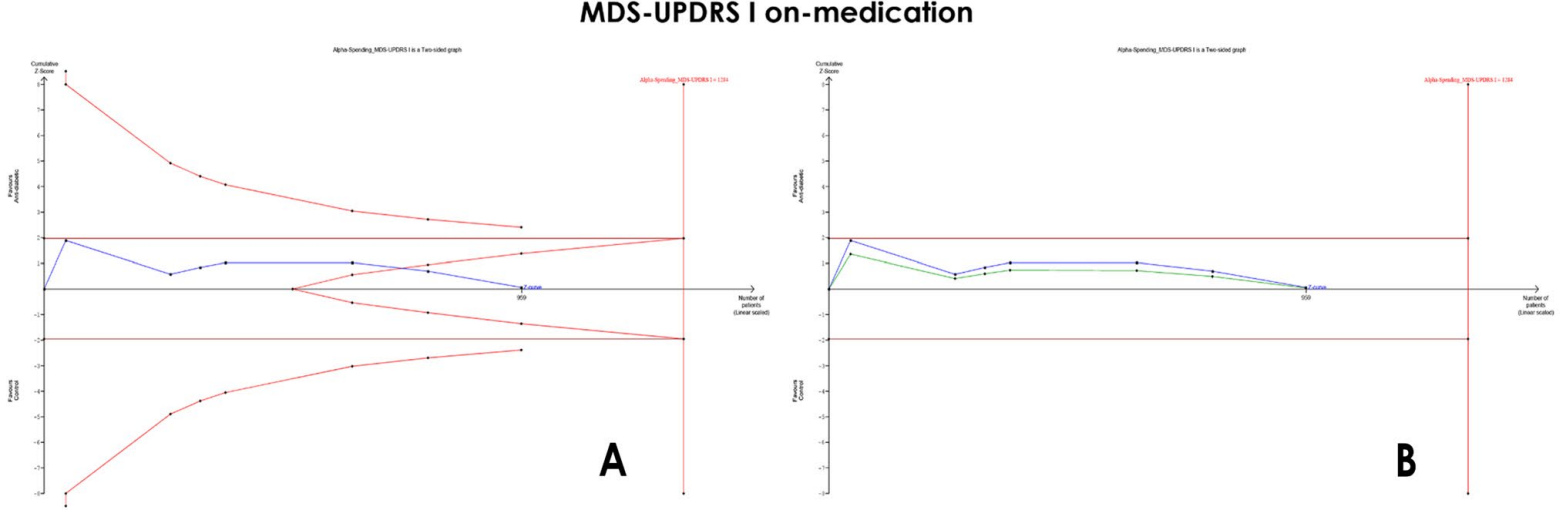
TSA on mean differences (MD) of MDS-UPDRS I (on medication). **A** MDS-UPDRS I cumulative *z*-curve passing the futility boundary (true negative). **B** MDS-UPDRS I penalized *Z*-curve not passing the conventional boundary

*MDS-UPDRS part I *(*on medication*) Our analysis revealed no significant difference between the two groups (MD = −0.04, 95% CI [−0.74 to 0.66], *p* = 0.90), with significant heterogeneity (*I*^2^ = 58%, *p* = 0.03) (Fig. 3A). The *α*-spending function adjusted the CI to (−0.61 to 0.58) indicating a non-significant pooled MD. The required information size of 1284 was not reached; however, the final point on the cumulative *z*-curve passed the futility boundary (true-negative region), indicating a conclusive result (Fig. 4A). Moreover, the penalized *Z*-curve did not pass the conventional boundary of (*z* = 1.96) (Fig. 4B).

**Fig. 5:**
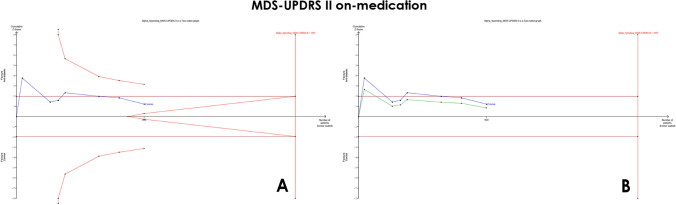
TSA on mean differences (MD) of MDS-UPDRS II (on medication), **A** MDS-UPDRS II cumulative *z*-curve not passing the superiority boundary nor the conventional boundary (False negative). **B** MDS-UPDRS II penalized *Z*-curve not passing the conventional boundary

*MDS-UPDRS part II* (*on medication*) Similarly, our results showed no significant difference between the two groups (MD = −0.88, 95% CI [−2.11 to 0.34], *p* = 0.16), with significant heterogeneity (*I*^2^ = 82%, *p* < 0.00001) (Fig. 3B). The *α*-spending adjusted CI to (−2.11 to 0.95) indicating a non-significant pooled MD. The final point on the cumulative *z*-curve did not pass the superiority monitoring boundary nor the conventional boundaries (false-negative region) indicating a non-conclusive result (Fig. 5A). In addition, the penalized *Z*-curve did not pass the conventional boundary of (*z* = 1.96) (Fig. 5B). The required information size of 2095 was not reached.

**Fig. 6:**
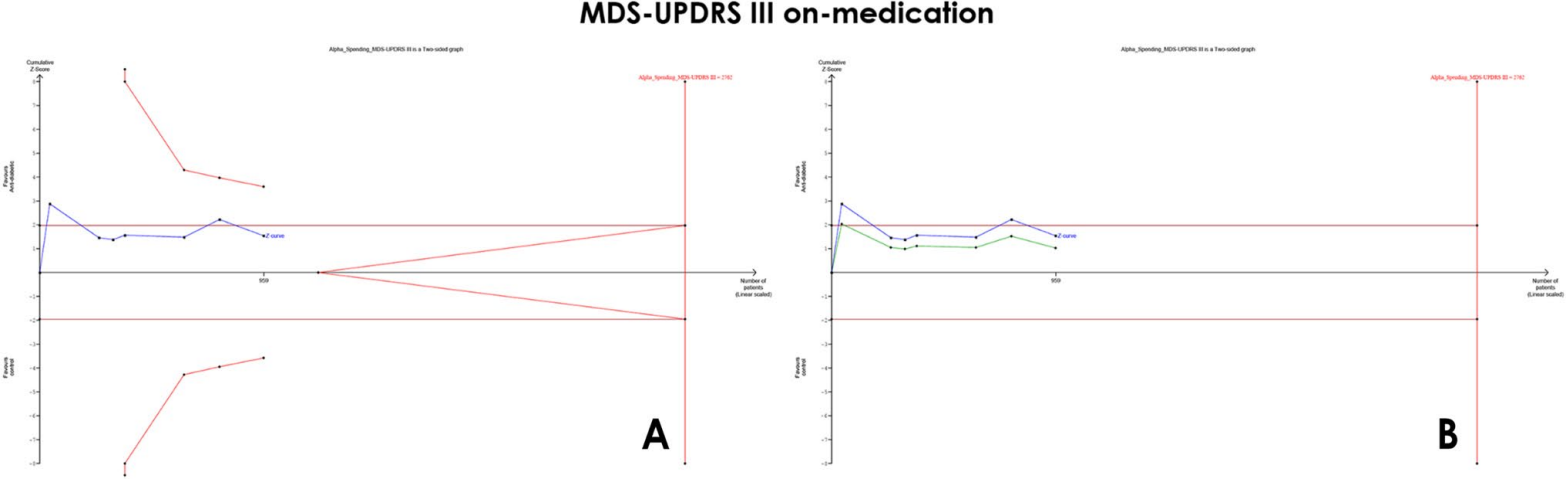
TSA on mean differences (MD) of MDS-UPDRS III (on medication) **A** MDS-UPDRS III cumulative *z*-curve not passing the superiority boundary nor the conventional boundary (False negative). **B** MDS-UPDRS III penalized *Z*-curve not passing the conventional boundary

*MDS-UPDRS part III* (*on medication*) Furthermore, our results showed no significant difference between the two groups (MD = −1.10, 95% CI [−2.61 to 0.42], *p* = 0.16), with significant heterogeneity (*I*^2^ = 60%, *p* = 0.02) (Fig. 3C). The *α*-spending adjusted CI (−3.37 to 1.34) indicates a non-significant pooled MD. Moreover, the final point on the cumulative *z*-curve did not pass either the superiority monitoring boundary or the conventional boundaries (false-negative region) indicating a non-conclusive (Fig. 6A). The penalized *Z*-curve did not pass the conventional boundary (*z* = 1.96) (Fig. 6B). The required information size of 2762 was not reached.

*MDS-UPDRS part IV* (*on medication*) Moreover, our results showed no significant difference between the two groups (MD = −0.09, 95% CI [−0.45 to 0.27], *p* = 0.64), with no heterogeneity among the pooled results (*I*^2^ = 3%, *p* = 0.39) (Fig. 3D). The last point on the cumulative *z*-curve did not surpass the traditional boundaries (*z* = 1.96). In addition, the penalized *Z*-curve did not pass the conventional boundary. However, the sequential monitoring boundary for the adjusted significance threshold was ignored due to too little information used (0.0%) (Supplementary Fig. 1).

*MDS-UPDRS part III* (*off medication*) As mentioned before, MDS-UPDRS part III was assessed while patients were off their conventional PD treatment; however, no significant difference was found between the two groups (MD = −1.43, 95% CI [−4.29 to 1.44], *p* = 0.33), with moderate heterogeneity (*I*^2^ = 69%, *p* = 0.01) (Fig. 3E). Similarly, the last point on the cumulative *z*-curve did not surpass the traditional boundaries (*z* = 1.96). In addition, the penalized *Z*-curve did not pass the conventional boundary. However, trials were ignored in interim due to too little information used (<1) (Supplementary Fig. 2).

#### Change from baseline in NMSS

**Fig. 7:**
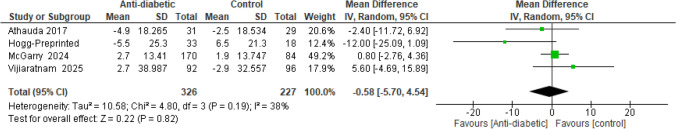
Forest plot comparing (MD) for change from baseline in NMSS

Our results showed no statistically significant difference between the two groups (MD = −0.58, 95% CI [−5.70 to 4.54], *p* = 0.82), with no heterogeneity (*I*^2^ = 38%, *p* = 0.19) (Fig. 7).

#### Change from baseline in PDQ-39 scores

**Fig. 8:**
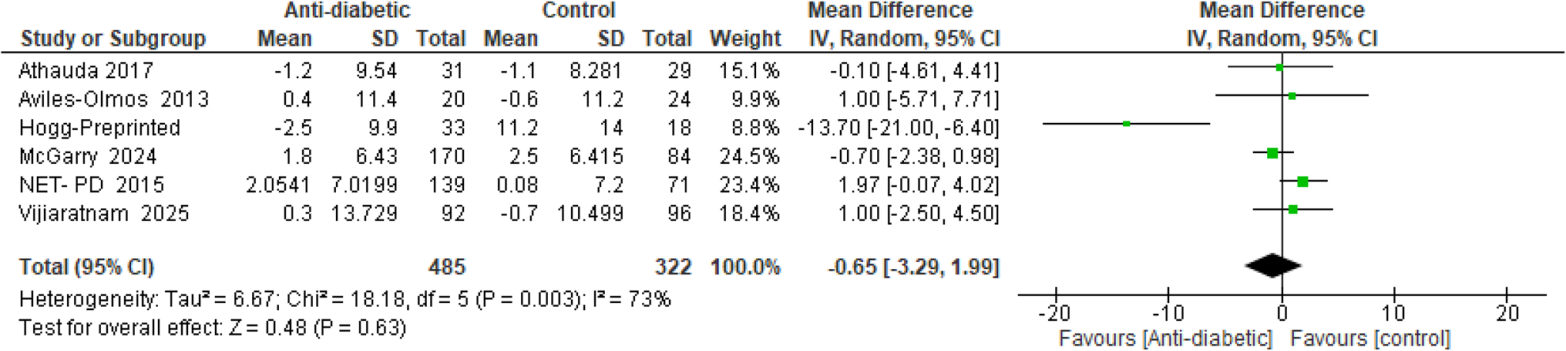
Forest plots comparing (MD) for change from baseline in PDQ-39

No significant difference was found between antidiabetic drugs and the control group (MD = −0.65, 95% CI [−3.29 to 1.99], *p* = 0.63), with significant heterogeneity (*I*^2^ = 73%, *p* = 0.003) (Fig. 8).

#### Change from baseline in MATTIS-DRS

**Fig. 9:**
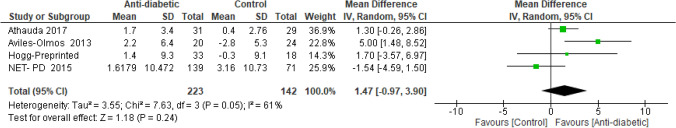
Forest plots comparing (MD) for change from baseline in MATTIS-DRS

Our results showed no statistically significant difference between the two groups (MD = 1.47, 95% CI [−0.97 to 3.90], *p* = 0.24), with significant heterogeneity (*I*^2^ = 61%, *p* = 0.05) (Fig. 9).

#### Change from baseline in MADRS

**Fig. 10:**
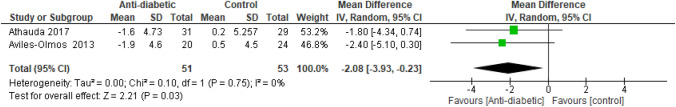
Forest plots comparing (MD) for change from baseline in MADRS

In contrast to all previous scales, our analysis revealed a statistically significant difference favoring antidiabetic drugs over the control group (MD = −2.08, 95% CI [−3.93 to −0.23], *p* = 0.03), with no heterogeneity (*I*^2^ = 0%, *p* = 0.75) (Fig. 10).

### Safety outcomes

**Fig. 11:**
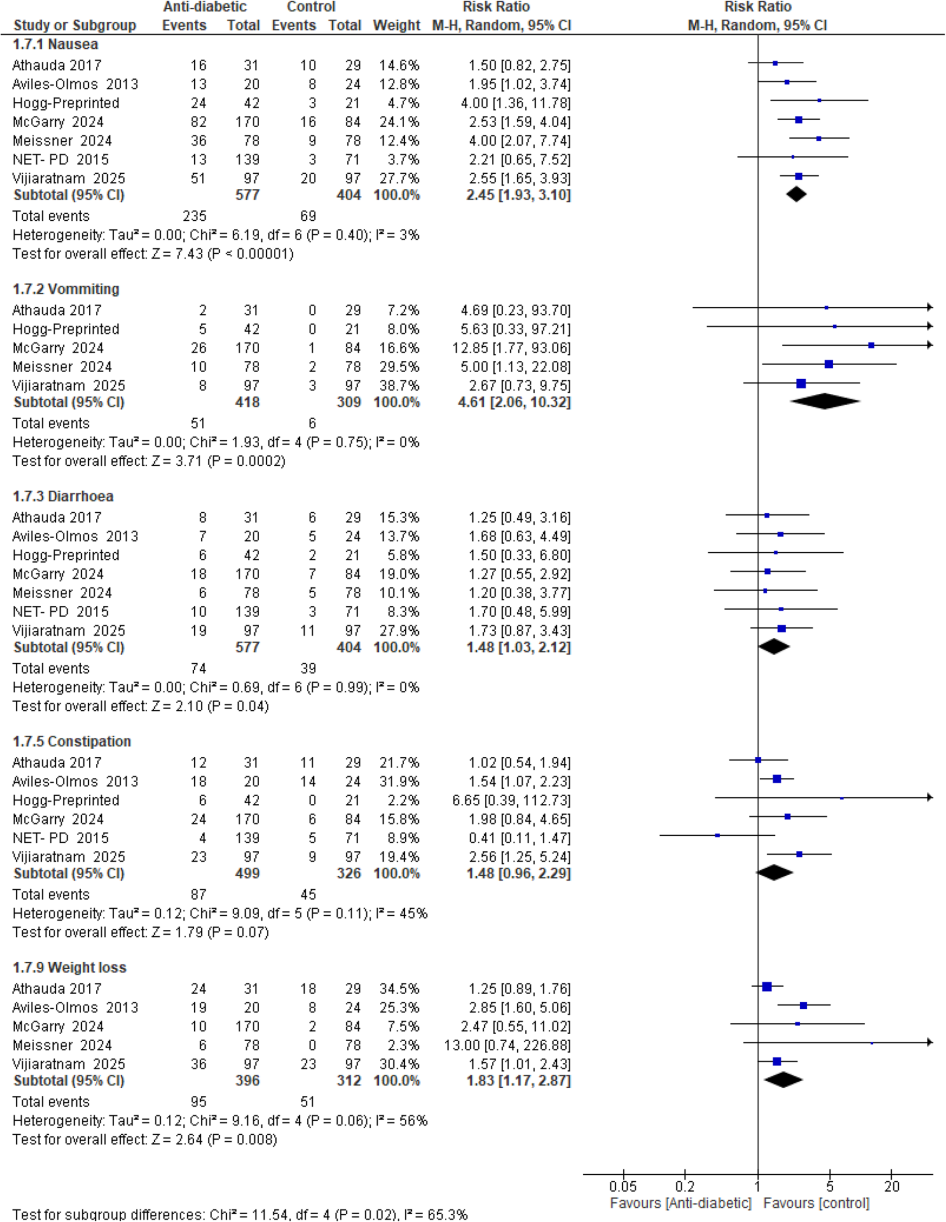
Forest plots comparing (RR) adverse effects (AEs) (nausea, vomiting, diarrhea, constipation, weight loss)

In *AEs*, statistically significant differences were observed between antidiabetic drugs and placebo favoring the placebo regarding *nausea, vomiting, diarrhea, and weight loss* (RR = 2.45, 95% CI [1.93 to 3.10], *p* < 0.00001), (RR = 4.61, 95% CI [2.06 to 10.32], *p* = 0.0002), (RR = 1.48, 95% CI [1.03 to 2.12], *p* = 0.04) or (RR = 1.83, 95% CI [1.17 to 2.87], *p* = 0.008), respectively. However, results revealed no statistically significant difference favoring either of the two groups concerning *constipation* (RR = 1.48, 95% CI [0.96 to 2.29], *p* = 0.07) (Fig. 11).

### Sensitivity analysis

In our sensitivity analysis, we conducted four main evaluations. First, in order to test the robustness of our evidence, we conducted a leave-one-out sensitivity analysis in multiple scenarios, excluding one study in each scenario making sure that the results were not dependent on a single study. Revealing that on excluding the study Vijiaratnam et al., the outcome MDS-UPDRS III (on medication) becomes statistically significant favoring the antidiabetic drugs (Supplementary Fig. 3). In addition, leave-one-out sensitivity analysis revealed that on excluding the study NET.PD et al., the outcome MATTIS-DRS becomes statistically significant favoring the interventional group indicating potential efficacy of GLP-1 agonists in improving the dementia rating scale (Supplementary Fig. 4). Moreover, the sensitivity analysis identified Olmos et al. and Hogg et al. as the primary contributors of heterogeneity in our meta-analysis, mostly due to their high risk of bias. Lastly, we implemented a sensitivity analysis for our TSA using pooled empirical data from all included studies, revealing no significant differences in the results which supports the robustness of our findings in various analytical scenarios (Supplementary Figs. [5–9]).

### GRADE evaluation of evidence

Grade results and summary profile are demonstrated in Table 3, with a detailed domain assessment in Supplementary Table 1. Regarding primary outcomes, results demonstrated a very low to moderate level of confidence, mainly due to concerns in inconsistency and imprecision.

**Table 3:** Summary of primary outcomes with grade evaluation of evidence

Outcome	Follow up	Med. status	No. of studies	No. of patients	MD [± 95% CI] Grade evaluation	Anticipated absolute effects (95% CI)		*p* value	Heterogeneity assessment *I*^2^ [*p *value]
						Risk with placebo	Risk with antidiabetic drugs		
MDS-UPDRS part I	36 to 54 weeks	On	7 RCTs	959	MD = −0.04, 95% CI [−0.74 to 0.66] **⨁⨁◯◯** **Low**	The mean change from baseline MDS-UPDRS part I (on medication) was 0.67	MD 0.04 lower (0.74 lower to 0.66 higher)	*p* = 0.90	*I*^2^ = 58% [*p* = 0.03]
MDS-UPDRS part II	36 to 54 weeks	On	7 RCTs	959	MD =−0.88, 95% CI [−2.11 to 0.34] **⨁⨁◯◯** **Low**	The mean change from baseline MDS-UPDRS part II (on medication) was 1.61	MD 0.88 lower (2.11 lower to 0.34 higher)	*p* = 0.16	*I*^2^ = 82% [*p* < 0.00001]
MDS-UPDRS part III	36 to 54 weeks	On	7 RCTs	959	MD = −1.10, 95% CI [−2.61 to 0.42] **⨁⨁◯◯** **Low**	The mean change from baseline MDS-UPDRS part III (on medication) was 2.47	MD 1.1 lower (2.61 lower to 0.42 higher)	*p* = 0.16	*I*^2^ = 60% [*p* = 0.02]
MDS-UPDRS part IV	36 to 54 weeks	On	5 RCTs	495	MD = −0.09, 95% CI [−0.45 to 0.27] **⨁⨁⨁◯** **Moderate**	The mean change from baseline MDS-UPDRS part IV (on medication) was 0.32	MD 0.09 lower (0.45 lower to 0.27 higher)	*p* = 0.64	*I*^2^ = 3% [*p* = 0.39]
MDS-UPDRS part III	36 to 54 weeks	Off	5 RCTs	495	MD = −1.43, 95% CI [−4.29 to 1.44] **⨁◯◯◯** **Very low**	The mean change from baseline MDS-UPDRS part III (off medication) was 1.2	MD 1.43 lower (4.29 lower to 1.44 higher)	*p* = 0.33	*I*^2^ = 69% [*p* = 0.01]

## Discussion

This systematic review and meta-analysis was conducted to assess the efficacy and safety of antidiabetic medications for early- to mid-stage PD as based on the Hoehn and Yahr scale. The efficacy of oral antidiabetics was assessed using MDS-UPDRS, NMSS, PDQ-39, MATTIS-DRS, and MADRS. Safety was assessed by the occurrence of adverse events, such as nausea, vomiting, diarrhea, and weight loss. 973 patients with PD taking antidiabetics in the form of SC GLP-1 agonists or oral thiazolidinediones were identified from seven studies. Only the MADRS scale showed a statistically significant difference for antidiabetic drugs over the control group. Moreover, antidiabetic drugs were associated with a high risk of nausea, vomiting, diarrhea, and weight loss.

MDS-UPDRS part I is concerned with the assessment of non-motor experiences of daily living, such as cognitive impairment, hallucinations, depression, anxiety, apathy, and sleep problems (Gallagher et al. 2012). As pooled from all seven included studies, we found no significant differences between the antidiabetic drug group and the control group, which is consistent with a previous meta-analysis investigating only GLP-1 agonists in relation to the control (Messak et al. 2025). This is also consistent with a review investigating the efficacy of GLP-1 agonists on cognitive, psychotic, and anxiety disorders in different patient populations; no effect was found for almost all the identified clinical trials for these outcomes (Giorgi et al. 2025). The NMSS also assesses non-motor experiences in patients with PD. A strong correlation was observed between the MDS-UPDRS part I total score and NMSS total score (Wamelen et al. 2021). Moreover, this association was found to be stronger for mild and moderate non-motor symptom severity and became weaker as the symptoms became more severe (Martinez-Martin et al. 2015). In our analysis, pooled from four studies using GLP-1 agonists, no significant differences were observed when compared to the control group. GLP-1 agonists have neuroprotective effects by reducing cytokine production, creating an anti-inflammatory environment, and reducing oxidative stress (Siddeeque et al. 2024). Moreover, preclinical studies have shown that they can protect synaptic numbers and dopaminergic neurons (Hölscher 2018; Dierssen and Barone 2021). The observed non-significant differences in both the MDS-UPDRS part I and NMSS could be influenced by the duration of the included trials. Moreover, the scope of non-motor symptoms is wide; thus, if GLP-1 agonists showed improvements in one domain, it could be masked by other non-motor manifestations. TSA was done regarding MDS-UPDRS part I. As the cumulative *z*-curve passed the futility boundaries, it is doubtful that future studies investigating non-motor symptoms would be able to demonstrate statistical significance (Nair and Borkar 2024). Cognitive impairment as a non-motor experience of PD can be assessed separately using the MATTIS-DRS. Non-significant differences were also found in cognitive function, as pooled from four studies. Although sensitivity analysis revealed a statistically significant difference favoring GLP-1 agonists. However, cognitive scales have been identified as non-substitutes for comprehensive neuropsychological tests (Skorvanek et al. 2018).

The MDS-UPDRS Part II assesses motor experiences of daily living, with scores significantly increasing with PD duration and severity (Rodriguez-Blazquez et al. 2013). No significant differences were found in the mean MDS-UPDRS Part II scores as pooled from all the seven included studies. Patients with PD develop nigrostriatal dopaminergic depletion with routine dopaminergic medications, such as levodopa and dopamine agonists, enhancing dopamine bioavailability in the brain (Woitalla et al. 2023; Meder et al. 2019). The absence of a significant difference in motor function could be a result of GLP-1 agonists not directly modulating dopamine levels, but enhancing autophagy and clearance of aggregated proteins and suppressing inflammation and microglial activation (Athauda and Foltynie 2016). Moreover, patients in both the GLP-1 agonist and control groups could be optimally treated with dopaminergic drugs that could mask any potential benefits of other drugs. This finding is similar to that of a previous meta-analysis, in which PD patients treated with exenatide or pioglitazone showed no significant differences in the MDS-UPDRS Part II (Wang et al. 2020). As the required information size in TSA was not reached for this outcome, the pooled estimate remains inconclusive, highlighting the need for future trials to confirm the absence of treatment effects.

The MDS-UPDRS Part III and MDS-UPDRS Part IV are also concerned with motor aspects of PD, assessing clinician-rated motor examination and occurrence of motor complications, respectively (Martinez-Martin et al. 2013; Raciti et al. 2019). No significant differences were found between the antidiabetic drugs and the control group, as pooled from seven studies for motor examination and five studies for motor complications. This may be a result of the included trials including only PD patients with early- to mid-stage disease progression, with most of the patients being in either Hoehn and Yahr stage 1 or 2. Moreover, there were variations in PD duration across studies. As motor complications, such as dyskinesia and motor fluctuations, are related to long-term therapy with levodopa, the short durations of some of the included trials may not be sufficient to detect an effect (Matarazzo et al. 2018; Biase et al. 2023). We also found no significant difference for MDS-UPDRS Part III, while patients were off their conventional PD treatment, as pooled from five studies. For all MDS-UPDRS Part III and MDS-UPDRS Part IV outcomes, the cumulative *z*-curve did not pass the conventional boundaries or futility boundaries, indicating that the results are still not conclusive.

The MADRS assesses the severity of depression in different neurological conditions, with high diagnostic utility in patients with PD (Ketharanathan et al. 2016). Two of the included studies provided MADRS data, and exenatide was used in both. Pooling the two studies showed a statistically significant difference favoring exenatide. This observation is similar to that of a previous study investigating the possible role of GLP-1 agonists as antidepressants. Patients with depression reported a decline in depression rates when receiving GLP-1 agonists compared to the control group (Chen et al. 2024). The exact mechanism underlying this effect remains unclear; however, depression is associated with brain insulin resistance, and GLP-1 agonists have been found to prevent insulin receptor loss in the brain (Hamer et al. 2019; Lyra e Silva et al. 2019; Batista et al. 2018). Another possible explanation is that GLP-1 agonists can augment serotonergic transmission, which is essential for mood processing and emotional regulation (Kim et al. 2020). Pooled from six studies assessing the impact of antidiabetic drugs on the quality of life of PD patients using the PDQ-39 scale, no significant difference was observed. This finding is similar to those of two recent meta-analyses investigating GLP-1 agonists for PD patients (Messak et al. 2025; Albuquerque et al. 2025). This highlights the complexity of PD and its multifaceted symptomologies, as while drugs could provide specific benefits, they may not address all aspects of a disease to impact quality of life, highlighting the need for a more holistic approach in PD management (Bloem et al. 2021).

Regarding safety outcomes, a statistically significant difference was observed for nausea, vomiting, diarrhea, and weight loss, favoring the control group. The onset of gastrointestinal side effects has been shown to be related to the initiation and up-titration of GLP-1 agonists, suggesting a potential dose-dependent effect, with nausea present in up to half of the treated patients (Wan et al. 2024; Liu et al. 2022). Fluctuating levels of the drug in short-acting formulations, in addition to their effects on delaying gastric emptying, have been suggested as possible mechanisms of nausea (Pratley et al. 2014; Nauck et al. 2011). The mechanisms underlying vomiting and diarrhea are not well understood but could be due to the effects of treatment on intestinal motility, the central nervous system, gastric emptying, and nutrient absorption (Wan et al. 2024). GLP-1 agonists reduce body weight secondary to the effects of reduced food intake in addition to delaying gastric emptying (Drucker 2022).

## Strengths

In this meta-analysis, we provide up-to-date evidence regarding the efficacy and safety of antidiabetic drugs in patients with PD. The analysis was based only on RCTs and followed the Cochrane Handbook for Systematic Reviews and Meta-Analyses and PRISMA guidelines. TSA was used to account for the risk of false-positive results and ensure the validity of our results. In addition, the TSA indicated that the required information size was not reached for multiple outcomes, highlighting the need for more trials to draw definitive conclusions. Sensitivity analyses were conducted to identify studies responsible for heterogeneity. Lastly, efficacy outcomes were assessed using multiple scales that were previously validated for PD.

## Limitations and implications for future research

Despite offering comprehensive evidence regarding the safety and efficacy of antidiabetic drugs in patients with PD, several limitations of this study should be acknowledged. There are variations in antidiabetic drugs and their doses across studies, in addition to the variability in trial durations that could affect the appropriate detection of treatment effects. The PD duration was also subject to variability across the studies. All of these could be the underlying cause for the significant heterogeneity in different measures, including the MDS-UPDRS part I, part II, part III (on medication), PDQ-39, and MATTIS-DRS. TSA results were inconclusive for most of the assessed outcomes. Lastly, only one of the included trials used an antidiabetic drug other than GLP-1 agonists, which could affect the generalizability of our results to all antidiabetic drugs. Further studies are needed to investigate the efficacy and safety of antidiabetic drugs other than GLP-1 agonists in patients with PD. We also recommend the use of objective rather than subjective measures to assess drug efficacy. Future research could also expand beyond the RCTs to minimize selection bias and achieve a better understanding. As we only included patients with mild-to-moderate PD, future research on patients with moderate-to-severe PD is needed to identify the treatment effect across all ranges of severity.

## Conclusion

This meta-analysis provides comprehensive evidence regarding the safety and efficacy of antidiabetic drugs in early-to mid-stage PD. Antidiabetic drugs showed no significant effect on non-motor symptoms, as detected by the MDS-UPDRS part I with TSA suggesting that this finding is conclusive. Antidiabetic drugs had no significant effect on motor experiences, motor examination, and motor complications of PD, and future trials are needed to confirm this finding according to TSA. A potential antidepressant effect of GLP-1 agonists has been found in PD patients. PD patients treated with antidiabetic drugs were at a higher risk for nausea, vomiting, diarrhea, and weight loss, aligning with their known gastrointestinal side effects. Future research should further explore the efficacy of antidiabetic drugs on the motor symptoms of PD, investigate PD patients with higher severity, and assess the efficacy and safety of other non-GLP-1 agonist antidiabetic drugs.

## Supplementary Information

Below is the link to the electronic supplementary material.

Supplementary file1 (PDF 13531 KB)

## Data Availability

The datasets used and/or analyzed during the current study are available from the corresponding author on reasonable request.

## References

[CR1] Abu-Hamdah R, Rabiee A, Meneilly GS, Shannon RP, Andersen DK, Elahi D (2009) The extrapancreatic effects of glucagon-like peptide-1 and related peptides. J Clin Endocrinol Metab 94:1843–1852 19336511 10.1210/jc.2008-1296 PMC2690432

[CR2] Albuquerque MB de, Nunes LEDB, Oliveira Maldonado JV de, Melo Ferreira DG, Margato MM, Rabelo LV, Valença MM, Oliveira Cordeiro LH (2025) GLP‐1 receptor agonists for Parkinson’s disease: an updated meta-analysis. Parkinsonism Relat Disord. 10.1016/j.parkreldis.2024.107220 10.1016/j.parkreldis.2024.107220 39642803

[CR3] Armstrong MJ, Okun MS (2020) Diagnosis and treatment of Parkinson disease. JAMA 323:548 32044947 10.1001/jama.2019.22360

[CR60] Athauda D et al (2017) Exenatide once weekly versus placebo in Parkinson’s disease: a randomised, double-blind, placebocontrolled trial. Lancet (London, England) 390(10103):1664–1675. 10.1016/S0140-6736(17)31585-4 28781108 10.1016/S0140-6736(17)31585-4 PMC5831666

[CR4] Athauda D, Foltynie T (2016) The glucagon-like peptide 1 (GLP) receptor as a therapeutic target in Parkinson’s disease: mechanisms of action. Drug Discov Today 21:802–818 26851597 10.1016/j.drudis.2016.01.013

[CR62] Aviles-Olmos I et al (2013) Exenatide and the treatment of patients with Parkinson’s disease. J Clin Invest 123(6):2730–2736. 10.1172/JCI68295 23728174 10.1172/JCI68295 PMC3668846

[CR5] Batista AF, Forny-Germano L, Clarke JR et al (2018) The diabetes drug liraglutide reverses cognitive impairment in mice and attenuates insulin receptor and synaptic pathology in a non-human primate model of Alzheimer’s disease. J Pathol 245:85–100 29435980 10.1002/path.5056 PMC5947670

[CR6] di Biase L, Pecoraro PM, Carbone SP, Caminiti ML, Di Lazzaro V (2023) Levodopa-induced dyskinesias in parkinson’s disease: an overview on pathophysiology, clinical manifestations, therapy management strategies and future directions. J Clin Med. 10.3390/jcm12134427 10.3390/jcm12134427 PMC10342913 37445461

[CR7] Bloem BR, Okun MS, Klein C (2021) Parkinson’s disease. The Lancet 397:2284–2303 10.1016/S0140-6736(21)00218-X 33848468

[CR8] Brás IC, Outeiro TF (2021) Alpha-synuclein: mechanisms of release and pathology progression in synucleinopathies. Cells 10:375 33673034 10.3390/cells10020375 PMC7917664

[CR9] Chen X, Zhao P, Wang W, Guo L, Pan Q (2024) The antidepressant effects of GLP-1 receptor agonists: a systematic review and meta-analysis. Am J Geriatr Psychiatry 32:117–127 37684186 10.1016/j.jagp.2023.08.010

[CR10] Cullinane PW, de Pablo FE, König A, Outeiro TF, Jaunmuktane Z, Warner TT (2023) Type 2 diabetes and Parkinson’s disease: a focused review of current concepts. Mov Disord 38:162–177 36567671 10.1002/mds.29298

[CR11] Cumpston M, Li T, Page MJ, Chandler J, Welch VA, Higgins JP, Thomas J (2019) Updated guidance for trusted systematic reviews: a new edition of the Cochrane Handbook for Systematic Reviews of Interventions. Cochrane Database Syst Rev ED000142 10.1002/14651858.ED000142 PMC10284251 31643080

[CR12] De Giorgi R, Ghenciulescu A, Dziwisz O et al (2025) An analysis on the role of glucagon-like peptide-1 receptor agonists in cognitive and mental health disorders. Nature Mental Health. 10.1038/s44220-025-00390-x 10.1038/s44220-025-00390-x PMC7619120 42239616

[CR13] de Lau LML, Breteler MMB (2006) Epidemiology of Parkinson’s disease. Lancet Neurol 5:525–535 16713924 10.1016/S1474-4422(06)70471-9

[CR14] Dierssen M, Barone E (2021) Editorial: brain insulin resistance in neurodevelopmental and neurodegenerative disorders: mind the gap! Front Neurosci. 10.3389/fnins.2021.730378 34447295 10.3389/fnins.2021.730378 PMC8382942

[CR15] Diz-Chaves Y, Mastoor Z, Spuch C, González-Matías LC, Mallo F (2022) Anti-inflammatory effects of GLP-1 receptor activation in the brain in neurodegenerative diseases. Int J Mol Sci 23:9583 36076972 10.3390/ijms23179583 PMC9455625

[CR16] Drucker DJ (2022) GLP-1 physiology informs the pharmacotherapy of obesity. Mol Metab. 10.1016/j.molmet.2021.101351 34626851 10.1016/j.molmet.2021.101351 PMC8859548

[CR17] Ferreira SA, Romero-Ramos M (2018) Microglia response during Parkinson’s disease: alpha-synuclein intervention. Front Cell Neurosci. 10.3389/fncel.2018.00247 30127724 10.3389/fncel.2018.00247 PMC6087878

[CR18] Gallagher DA, Goetz CG, Stebbins G, Lees AJ, Schrag A (2012) Validation of the MDS-UPDRS Part I for nonmotor symptoms in Parkinson’s disease. Mov Disord 27:79–83 21915909 10.1002/mds.23939

[CR19] Garcia P, Jürgens-Wemheuer W, Huarte OU et al (2022) Neurodegeneration and neuroinflammation are linked, but independent of alpha-synuclein inclusions, in a seeding/spreading mouse model of Parkinson’s disease. Glia 70:935–960 35092321 10.1002/glia.24149 PMC9305192

[CR20] Grieco M, Giorgi A, Gentile MC, d’Erme M, Morano S, Maras B, Filardi T (2019) Glucagon-like peptide-1: a focus on neurodegenerative diseases. Front Neurosci. 10.3389/fnins.2019.01112 31680842 10.3389/fnins.2019.01112 PMC6813233

[CR21] Hamer JA, Testani D, Mansur RB, Lee Y, Subramaniapillai M, McIntyre RS (2019) Brain insulin resistance: a treatment target for cognitive impairment and anhedonia in depression. Exp Neurol 315:1–8 30695707 10.1016/j.expneurol.2019.01.016

[CR57] Hogg E et al (2022) A phase II, randomized, double-blinded, placebo-controlled trial of liraglutide in Parkinson’s disease. SSRN Preprint. 10.2139/ssrn.4212371

[CR22] Hölscher C (2018) Novel dual GLP-1/GIP receptor agonists show neuroprotective effects in Alzheimer’s and Parkinson’s disease models. Neuropharmacology 136:251–259 29402504 10.1016/j.neuropharm.2018.01.040

[CR23] Ketharanathan T, Hanwella R, Weerasundera R, De Silva VA (2016) Diagnostic validity and factor analysis of Montgomery-Asberg depression rating scale in Parkinson disease population. J Geriatr Psychiatry Neurol 29:115–119 26392481 10.1177/0891988715606232

[CR24] Kim YK, Kim OY, Song J (2020) Alleviation of depression by glucagon-like Peptide 1 through the regulation of neuroinflammation, neurotransmitters, neurogenesis, and synaptic function. Front Pharmacol. 10.3389/fphar.2020.01270 32922295 10.3389/fphar.2020.01270 PMC7456867

[CR25] Leon N, LaCoursiere R, Yarosh D, Patel RS (2017) Lixisenatide (Adlyxin): a once-daily incretin mimetic injection for type-2 diabetes. P T 42:676–711 29089722 PMC5642155

[CR26] Liu L, Chen J, Wang L, Chen C, Chen L (2022) Association between different GLP-1 receptor agonists and gastrointestinal adverse reactions: a real-world disproportionality study based on FDA adverse event reporting system database. Front Endocrinol (Lausanne). 10.3389/fendo.2022.1043789 36568085 10.3389/fendo.2022.1043789 PMC9770009

[CR27] Lovshin JA, Drucker DJ (2009) Incretin-based therapies for type 2 diabetes mellitus. Nat Rev Endocrinol 5:262–269 19444259 10.1038/nrendo.2009.48

[CR28] Lyra e Silva N de M, Lam MP, Soares CN, Munoz DP, Milev R, De Felice FG (2019) Insulin resistance as a shared pathogenic mechanism between depression and type 2 diabetes. Front Psychiatry. 10.3389/fpsyt.2019.00057 10.3389/fpsyt.2019.00057 PMC6382695 30837902

[CR29] Martinez-Martin P, Rodriguez-Blazquez C, Alvarez-Sanchez M et al (2013) Expanded and independent validation of the Movement Disorder Society-Unified Parkinson’s Disease Rating Scale (MDS-UPDRS). J Neurol 260:228–236 22865238 10.1007/s00415-012-6624-1

[CR30] Martinez-Martin P, Chaudhuri KR, Rojo-Abuin JM et al (2015) Assessing the non-motor symptoms of Parkinson’s disease: MDS-UPDRS and NMS Scale. Eur J Neurol 22:37–43 23607783 10.1111/ene.12165

[CR31] Matarazzo M, Perez-Soriano A, Stoessl AJ (2018) Dyskinesias and levodopa therapy: why wait? J Neural Transm 125:1119–1130 29428995 10.1007/s00702-018-1856-6

[CR61] McGarry A et al (2024) Safety, tolerability, and efficacy of NLY01 in early untreated Parkinson’s disease: a randomised, doubleblind, placebo-controlled trial. Lancet Neurol 23(1):37–45. 10.1016/S1474-4422(23)00378-2 38101901 10.1016/S1474-4422(23)00378-2

[CR32] Meder D, Herz DM, Rowe JB, Lehéricy S, Siebner HR (2019) The role of dopamine in the brain—lessons learned from Parkinson’s disease. Neuroimage 190:79–93 30465864 10.1016/j.neuroimage.2018.11.021

[CR59] Meissner WG et al (2024) Trial of Lixisenatide in Early Parkinson’s Disease. N Engl J Med 390(13):1176–1185. 10.1056/NEJMoa2312323 38598572 10.1056/NEJMoa2312323

[CR33] Messak M, Abdelmageed A, Senbel AA et al (2025) Efficacy and safety of GLP-1 agonists in Parkinson’s disease: a systematic review and meta-analysis of randomized controlled trials. Naunyn Schmiedebergs Arch Pharmacol. 10.1007/s00210-025-03932-3 40067438 10.1007/s00210-025-03932-3 PMC12350457

[CR34] Moreno S, Farioli-Vecchioli S, Cerù MP (2004) Immunolocalization of peroxisome proliferator-activated receptors and retinoid X receptors in the adult rat CNS. Neuroscience 123:131–145 14667448 10.1016/j.neuroscience.2003.08.064

[CR35] Nair A, Borkar NK (2024) Understanding the trial sequential analysis graph in meta-analysis. Saudi J Anaesth 18:162–164 38313741 10.4103/sja.sja_715_23 PMC10833018

[CR36] Nauck MA, Kemmeries G, Holst JJ, Meier JJ (2011) Rapid tachyphylaxis of the glucagon-like Peptide 1—induced deceleration of gastric emptying in humans. Diabetes 60:1561–1565 21430088 10.2337/db10-0474 PMC3292331

[CR37] Nicolakakis N, Aboulkassim T, Ongali B, Lecrux C, Fernandes P, Rosa-Neto P, Tong X-K, Hamel E (2008) Complete rescue of cerebrovascular function in aged Alzheimer’s disease transgenic mice by antioxidants and pioglitazone, a peroxisome proliferator-activated receptor gamma agonist. J Neurosci 28:9287–9296 18784309 10.1523/JNEUROSCI.3348-08.2008 PMC6670922

[CR58] NINDS Exploratory Trials in Parkinson Disease (NET-PD) FS-ZONE Investigators (2015) Pioglitazone in early Parkinson’s disease: a phase 2, multicentre, double-blind, randomised trial. Lancet Neurol 14(8):795–803. 10.1016/S1474-4422(15)00144-1 26116315 10.1016/S1474-4422(15)00144-1 PMC4574625

[CR38] Olanow CW, Kieburtz K, Schapira AHV (2009) Why have we failed to achieve neuroprotection in Parkinson’s disease? Ann Neurol 64:S101–S110 10.1002/ana.21461 19127580

[CR39] Ono K (2017) The oligomer hypothesis in α-synucleinopathy. Neurochem Res 42:3362–3371 28828740 10.1007/s11064-017-2382-x

[CR40] Ouzzani M, Hammady H, Fedorowicz Z, Elmagarmid A (2016) Rayyan-a web and mobile app for systematic reviews. Syst Rev 5:1–10 27919275 10.1186/s13643-016-0384-4 PMC5139140

[CR41] Page MJ, McKenzie JE, Bossuyt PM et al (2021) The PRISMA 2020 statement: an updated guideline for reporting systematic reviews. BMJ. 10.1136/BMJ.N71 33782057 10.1136/bmj.n71 PMC8005924

[CR42] Pratley RE, Nauck MA, Barnett AH et al (2014) Once-weekly albiglutide versus once-daily liraglutide in patients with type 2 diabetes inadequately controlled on oral drugs (HARMONY 7): a randomised, open-label, multicentre, non-inferiority phase 3 study. Lancet Diabetes Endocrinol 2:289–297 24703047 10.1016/S2213-8587(13)70214-6

[CR43] Raciti L, Nicoletti A, Mostile G, Bonomo R, Dibilio V, Donzuso G, Sciacca G, Cicero CE, Luca A, Zappia M (2019) Accuracy of MDS-UPDRS section IV for detecting motor fluctuations in Parkinson’s disease. Neurol Sci 40:1271–1273 30737581 10.1007/s10072-019-03745-2

[CR44] Rodriguez-Blazquez C, Rojo-Abuin JM, Alvarez-Sanchez M et al (2013) The MDS-UPDRS Part II (motor experiences of daily living) resulted useful for assessment of disability in Parkinson’s disease. Parkinsonism Relat Disord 19:889–893 23791519 10.1016/j.parkreldis.2013.05.017

[CR45] Rowlands J, Heng J, Newsholme P, Carlessi R (2018) Pleiotropic effects of GLP-1 and analogs on cell signaling, metabolism, and function. Front Endocrinol (Lausanne). 10.3389/fendo.2018.00672 30532733 10.3389/fendo.2018.00672 PMC6266510

[CR46] Schünemann HJ, Higgins JPT, Vist GE, Glasziou P, Akl EA, Skoetz N, Guyatt GH (2024) Chapter 14: Completing ‘Summary of findings’ tables and grading the certainty of the evidence [last updated August 2023]. In: Higgins JPT et al (eds.) Cochrane Handbook for Systematic Reviews of Interventions version 6.5. Cochrane

[CR47] Siddeeque N, Hussein MH, Abdelmaksoud A, Bishop J, Attia AS, Elshazli RM, Fawzy MS, Toraih EA (2024) Neuroprotective effects of GLP-1 receptor agonists in neurodegenerative disorders: a large-scale propensity-matched cohort study. Int Immunopharmacol. 10.1016/j.intimp.2024.113537 39486172 10.1016/j.intimp.2024.113537

[CR48] Skorvanek M, Goldman JG, Jahanshahi M et al (2018) Global scales for cognitive screening in Parkinson’s disease: critique and recommendations. Mov Disord 33:208–218 29168899 10.1002/mds.27233

[CR49] Sterne JAC, Savović J, Page MJ et al (2019) RoB 2: a revised tool for assessing risk of bias in randomised trials. BMJ. 10.1136/BMJ.L4898 31462531 10.1136/bmj.l4898

[CR50] Storer PD, Xu J, Chavis J, Drew PD (2005) Peroxisome proliferator-activated receptor-gamma agonists inhibit the activation of microglia and astrocytes: implications for multiple sclerosis. J Neuroimmunol 161:113–122 15748950 10.1016/j.jneuroim.2004.12.015

[CR51] Wetterslev J et al (2008) Trial sequential analysis may establish when firm evidence is reached in cumulative meta-analysis. J Clin Epidemiol 61(1):64–75. 10.1016/j.jclinepi.2007.03.013 18083463 10.1016/j.jclinepi.2007.03.013

[CR52] van Wamelen DJ, Martinez-Martin P, Weintraub D, Schrag A, Antonini A, Falup-Pecurariu C, Odin P, Ray Chaudhuri K (2021) The non-motor symptoms scale in Parkinson’s disease: validation and use. Acta Neurol Scand 143:3–12 32813911 10.1111/ane.13336

[CR63] Vijiaratnam N et al (2025) Exenatide once a week versus placebo as a potential disease-modifying treatment for people with Parkinson’s disease in the UK: a phase 3, multicentre, double-blind, parallel-group, randomised, placebo-controlled trial. Lancet (London, England) 405(10479):627–636. 10.1016/S0140-6736(24)02808-3 39919773 10.1016/S0140-6736(24)02808-3

[CR53] Wan J, Ferrari C, Tadros M (2024) GLP-1RA Essentials in gastroenterology: side effect management, precautions for endoscopy and applications for gastrointestinal disease treatment. Gastroenterol Insights 15:191–212

[CR54] Wang SY, Wu SL, Chen TC, Sen CC (2020) Antidiabetic agents for treatment of Parkinson’s disease: a meta-analysis. Int J Environ Res Public Health 17:1–11 10.3390/ijerph17134805 PMC7369739 32635358

[CR55] Woitalla D, Buhmann C, Hilker-Roggendorf R, Höglinger G, Koschel J, Müller T, Weise D (2023) Role of dopamine agonists in Parkinson’s disease therapy. J Neural Transm 130:863–873 37165120 10.1007/s00702-023-02647-0

[CR56] Zhang B, Song C, Tang X, Tian M, Liu Y, Yan Z, Duan R, Liu Y (2024) Type 2 diabetes microenvironment promotes the development of Parkinson’s disease by activating microglial cell inflammation. Front Cell Dev Biol 12:1422746 39050892 10.3389/fcell.2024.1422746 PMC11266050

