# Supplementary material for: Antidiabetic drugs in Parkinson’s disease: a comprehensive meta-analysis on efficacy and safety with trial sequential analysis and GRADE evaluation
Source: Inflammopharmacology. 2025 Aug 5;33(8):4577–93. doi: 10.1007/s10787-025-01873-0 (PMC12397177; doi:10.1007/s10787-025-01873-0)
Supplement: Supplementary file 1 — Supplementary file1 (PDF 13531 KB) [file 10787_2025_1873_MOESM1_ESM.pdf]

Supplementary table.1 Detailed Grade Evaluation of evidence domains.

Outcome	Risk of bias	Indirectness	inconsistency	Imprecision	Publication bias	GRADE Evaluation
**MDS-UPDRS part I**	No [a]	No	Yes [b]	Yes [c]	N/A	⊕⊕◯◯ **Low[a,b,c]**
**MDS-UPDRS part II**	No [a, d]	No	Yes [e]	Yes [f]	N/A	⊕⊕◯◯ **Low[a,d,e,f]**
**MDS-UPDRS part III**	No [a]	No	Yes [b]	Yes [f]	N/A	⊕⊕◯◯ **Low[a,b,f]**
**MDS-UPDRS part IV**	No [a]	No	No	Yes [f]	N/A	⊕⊕⊕◯ **Moderate[a,f]**
**MDS-UPDRS part III**	Yes [g]	No	Yes [e]	Yes [f]	N/A	⊕◯◯◯ **Very low[e,f,g]**

Explanations:

a. Some concerns in risk of bias about funding representatives participating in study design, analysis, and data interpretation. but not rated down for risk of bias.

b. Visual inconsistency confirmed with statistical analysis showing moderate heterogeneity.

c. Required information size wasn't reached, however cumulative z-curve passed the futility boundary suggesting sufficient imprecision. Moreover, the CI is wide including the line of no effect (including both benefit and harm).

d. The overall risk of bias across studies is relatively low with only 2 studies presenting a high risk contributing with about 20% of the weight of the analysis so not rated down for risk of bias.

e. Visual inconsistency confirmed with statistical analysis showing substantial heterogeneity.

f. TSA concluded that the required information size wasn't reached yet. Moreover, the CI is wide including the line of no effect (including both benefit and harm).

g. This outcome was influenced by contributions from all included studies risk of bias concerns collectively, additionally due to the contribution of high-risk bias studies with weight of 35%. Firstly, performance bias due to concerns in allocation concealment as well as involvement of the funders in data interpretation and study design which may have affected the reliability of the outcome as well as attrition bias concerns due to participants withdraws from some studies with the missing data not always equally distributed among the groups. Therefore, it was rated down for risk of bias.

attachment to manuscript

Click here to access/download;attachment to manuscript;Supplementary.docx

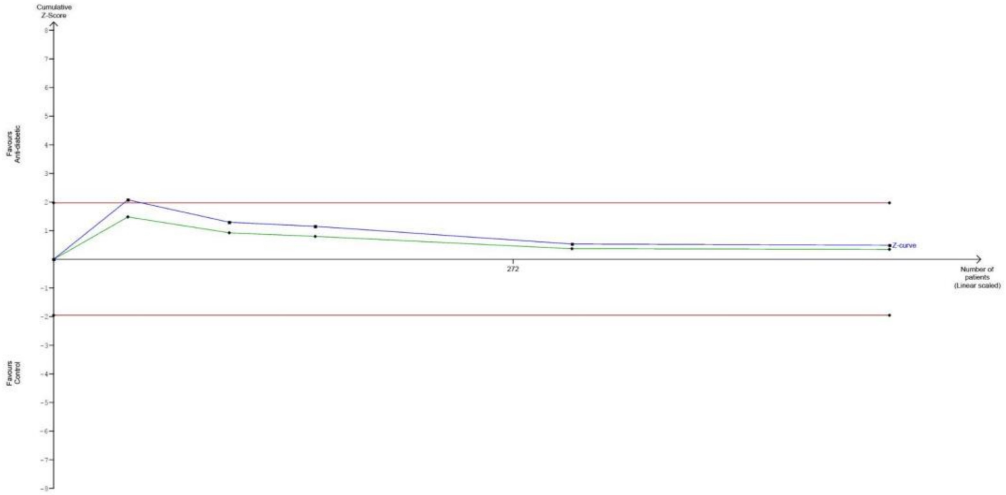

(Supplementary fig.1) TSA on MDS-UPDRS IV (on medication)

MDS-UPDRS IV penalized Z-curve not passing the conventional boundary

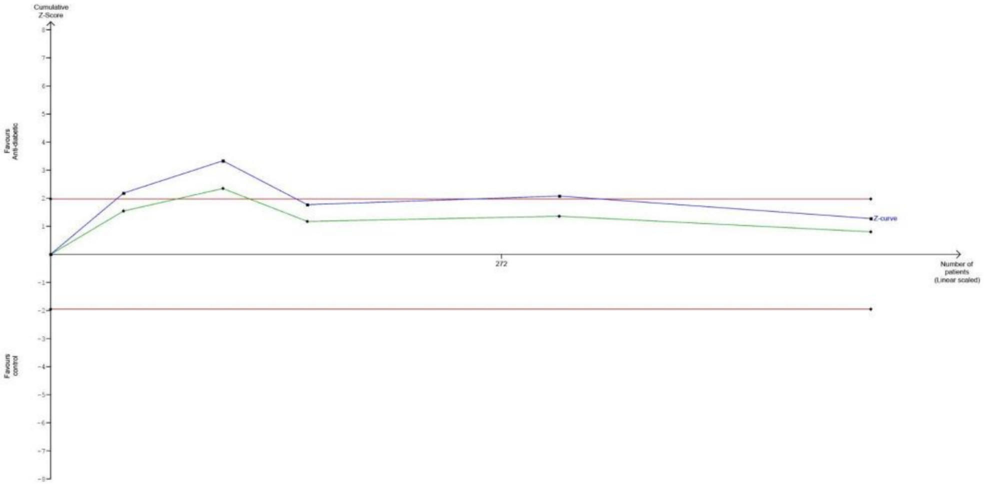

(Supplementary fig.2) TSA on MDS-UPDRS III (off medication)

MDS-UPDRS III penalized Z-curve not passing the conventional boundary

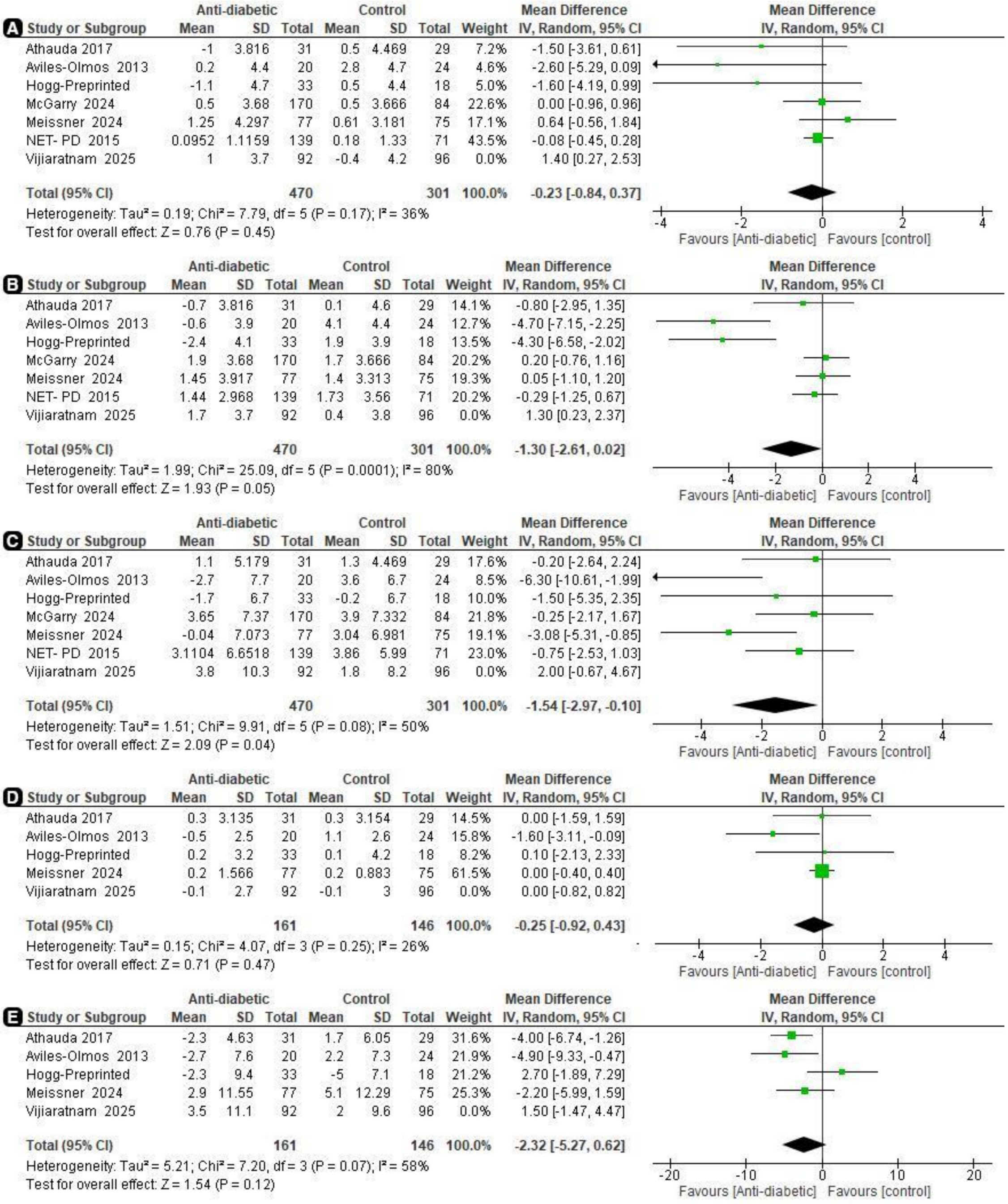

A

Study or Subgroup	Anti-diabetic Mean	Anti-diabetic SD	Anti-diabetic Total	Control Mean	Control SD	Control Total	Weight	Mean Difference IV, Random, 95% CI
Athauda 2017	-1	3.816	31	0.5	4.469	29	7.2%	-1.50 [-3.61, 0.61]
Aviles-Olmos 2013	0.2	4.4	20	2.8	4.7	24	4.6%	-2.60 [-5.29, 0.09]
Hogg-Preprinted	-1.1	4.7	33	0.5	4.4	18	5.0%	-1.60 [-4.19, 0.99]
McGarry 2024	0.5	3.68	170	0.5	3.666	84	22.6%	0.00 [-0.96, 0.96]
Meissner 2024	1.25	4.297	77	0.61	3.181	75	17.1%	0.64 [-0.56, 1.84]
NET- PD 2015	0.0952	1.1159	139	0.18	1.33	71	43.5%	-0.08 [-0.45, 0.28]
Vijiaratnam 2025	1	3.7	92	-0.4	4.2	96	0.0%	1.40 [0.27, 2.53]
**Total (95% CI)**			**470**			**301**	**100.0%**	**-0.23 [-0.84, 0.37]**

Heterogeneity: Tau² = 0.19; Chi² = 7.79, df = 5 (P = 0.17); I² = 36%
Test for overall effect: Z = 0.76 (P = 0.45)

B

Study or Subgroup	Anti-diabetic Mean	Anti-diabetic SD	Anti-diabetic Total	Control Mean	Control SD	Control Total	Weight	Mean Difference IV, Random, 95% CI
Athauda 2017	-0.7	3.816	31	0.1	4.6	29	14.1%	-0.80 [-2.95, 1.35]
Aviles-Olmos 2013	-0.6	3.9	20	4.1	4.4	24	12.7%	-4.70 [-7.15, -2.25]
Hogg-Preprinted	-2.4	4.1	33	1.9	3.9	18	13.5%	-4.30 [-6.58, -2.02]
McGarry 2024	1.9	3.68	170	1.7	3.666	84	20.2%	0.20 [-0.76, 1.16]
Meissner 2024	1.45	3.917	77	1.4	3.313	75	19.3%	0.05 [-1.10, 1.20]
NET- PD 2015	1.44	2.968	139	1.73	3.56	71	20.2%	-0.29 [-1.25, 0.67]
Vijiaratnam 2025	1.7	3.7	92	0.4	3.8	96	0.0%	1.30 [0.23, 2.37]
**Total (95% CI)**			**470**			**301**	**100.0%**	**-1.30 [-2.61, 0.02]**

Heterogeneity: Tau² = 1.99; Chi² = 25.09, df = 5 (P = 0.0001); I² = 80%
Test for overall effect: Z = 1.93 (P = 0.05)

C

Study or Subgroup	Anti-diabetic Mean	Anti-diabetic SD	Anti-diabetic Total	Control Mean	Control SD	Control Total	Weight	Mean Difference IV, Random, 95% CI
Athauda 2017	1.1	5.179	31	1.3	4.469	29	17.6%	-0.20 [-2.64, 2.24]
Aviles-Olmos 2013	-2.7	7.7	20	3.6	6.7	24	8.5%	-6.30 [-10.61, -1.99]
Hogg-Preprinted	-1.7	6.7	33	-0.2	6.7	18	10.0%	-1.50 [-5.35, 2.35]
McGarry 2024	3.65	7.37	170	3.9	7.332	84	21.8%	-0.25 [-2.17, 1.67]
Meissner 2024	-0.04	7.073	77	3.04	6.981	75	19.1%	-3.08 [-5.31, -0.85]
NET- PD 2015	3.1104	6.6518	139	3.86	5.99	71	23.0%	-0.75 [-2.53, 1.03]
Vijiaratnam 2025	3.8	10.3	92	1.8	8.2	96	0.0%	2.00 [-0.67, 4.67]
**Total (95% CI)**			**470**			**301**	**100.0%**	**-1.54 [-2.97, -0.10]**

Heterogeneity: Tau² = 1.51; Chi² = 9.91, df = 5 (P = 0.08); I² = 50%
Test for overall effect: Z = 2.09 (P = 0.04)

D

Study or Subgroup	Anti-diabetic Mean	Anti-diabetic SD	Anti-diabetic Total	Control Mean	Control SD	Control Total	Weight	Mean Difference IV, Random, 95% CI
Athauda 2017	0.3	3.135	31	0.3	3.154	29	14.5%	0.00 [-1.59, 1.59]
Aviles-Olmos 2013	-0.5	2.5	20	1.1	2.6	24	15.8%	-1.60 [-3.11, -0.09]
Hogg-Preprinted	0.2	3.2	33	0.1	4.2	18	8.2%	0.10 [-2.13, 2.33]
Meissner 2024	0.2	1.566	77	0.2	0.883	75	61.5%	0.00 [-0.40, 0.40]
Vijiaratnam 2025	-0.1	2.7	92	-0.1	3	96	0.0%	0.00 [-0.82, 0.82]
**Total (95% CI)**			**161**			**146**	**100.0%**	**-0.25 [-0.92, 0.43]**

Heterogeneity: Tau² = 0.15; Chi² = 4.07, df = 3 (P = 0.25); I² = 26%
Test for overall effect: Z = 0.71 (P = 0.47)

E

Study or Subgroup	Anti-diabetic Mean	Anti-diabetic SD	Anti-diabetic Total	Control Mean	Control SD	Control Total	Weight	Mean Difference IV, Random, 95% CI
Athauda 2017	-2.3	4.63	31	1.7	6.05	29	31.6%	-4.00 [-6.74, -1.26]
Aviles-Olmos 2013	-2.7	7.6	20	2.2	7.3	24	21.9%	-4.90 [-9.33, -0.47]
Hogg-Preprinted	-2.3	9.4	33	-5	7.1	18	21.2%	2.70 [-1.89, 7.29]
Meissner 2024	2.9	11.55	77	5.1	12.29	75	25.3%	-2.20 [-5.99, 1.59]
Vijiaratnam 2025	3.5	11.1	92	2	9.6	96	0.0%	1.50 [-1.47, 4.47]
**Total (95% CI)**			**161**			**146**	**100.0%**	**-2.32 [-5.27, 0.62]**

Heterogeneity: Tau² = 5.21; Chi² = 7.20, df = 3 (P = 0.07); I² = 58%
Test for overall effect: Z = 1.54 (P = 0.12)

(Supplementary fig.3) Sensitivity analysis to assess the impact of the study Vijiaratnam et al on the overall effect estimate in Change in MDS -UPDRS scores.

Study or Subgroup	Anti-diabetic Mean	SD	Total	Control Mean	SD	Total	Weight	Mean Difference IV, Random, 95% CI
Athauda 2017	1.7	3.4	31	0.4	2.76	29	55.3%	1.30 [-0.26, 2.86]
Aviles-Olmos 2013	2.2	6.4	20	-2.8	5.3	24	28.5%	5.00 [1.48, 8.52]
Hogg-Preprinted	1.4	9.3	33	-0.3	9.1	18	16.2%	1.70 [-3.57, 6.97]
NET- PD 2015	1.6179	10.472	139	3.16	10.73	71	0.0%	-1.54 [-4.59, 1.50]
**Total (95% CI)**			**84**			**71**	**100.0%**	**2.42 [0.01, 4.83]**

Heterogeneity: $Tau^2 = 2.10$; $Chi^2 = 3.56$, df = 2 (P = 0.17); $I^2 = 44\%$
Test for overall effect: Z = 1.97 (P = 0.05)

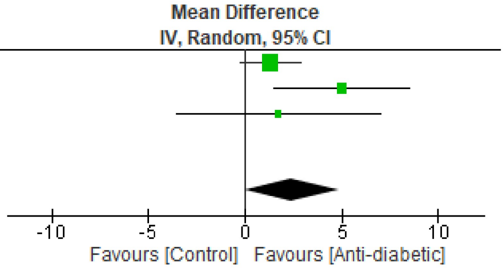

(Supplementary fig.4) Sensitivity analysis to assess the impact of the study NET-PD et al on the overall effect estimate of change in MATTIS DRS.

1. **TSA sensitivity analysis:**

To further test the robustness of our conclusions we conducted a sensitivity analysis on the same outcomes (MDS-UPDRS parts "I, II, III, IV" while patients were on conventional PD medications as well as MDS-UPDRS part III while off medications) employing the Biggerstaff-Tweedie (BT) random effects model. We used the pooled Empirical data from all the studies to form the monitoring boundaries.

1.1. MDS-UPDRS part I (on medication):

The last point on either the cumulative z-curve or the penalized z-curve did not surpass the traditional boundaries (z=1.96). However, the sequential monitoring boundary for the adjusted significance threshold was ignored due to too little information used (0.02%) **(Supplementary fig.5)**

1.2. MDS-UPDRS part II (on medication):

The final point on the cumulative z-curve didn't pass the conventional boundaries indicating a non-conclusive (False negative). Moreover, the penalized Z-curve didn't pass the conventional boundary (z=1.96). **(Supplementary fig.6)** However, trials were ignored in the interim due to too low information use.

1.3. MDS-UPDRS part III (on medication):

The final point on the cumulative z-curve didn't pass either the superiority monitoring boundary or the conventional boundaries indicating a non-conclusive (False negative). **(Supplementary fig.7A)** The penalized Z-curve didn't pass the conventional boundary (z=1.96). **(Supplementary fig.7B)** The required information size of 3188 wasn't reached.

1.4. MDS-UPDRS part IV (on medication):

The last point on either the cumulative z-curve or the penalized z-curve did not surpass the traditional boundaries (z=1.96). However, the sequential monitoring boundary for the adjusted significance threshold was ignored due to too little information used (2.63 %) **(Supplementary fig.8)**

1.5. MDS-UPDRS part III (off medication):

the final point on the cumulative z-curve didn't pass either the superiority monitoring boundary or the conventional boundaries indicating a non-conclusive (False negative). **(Supplementary fig.9A)** The

penalized Z-curve didn't pass the conventional boundary (z=1.96). **(Supplementary fig.9B)** The required information size of 2423 wasn't reached.

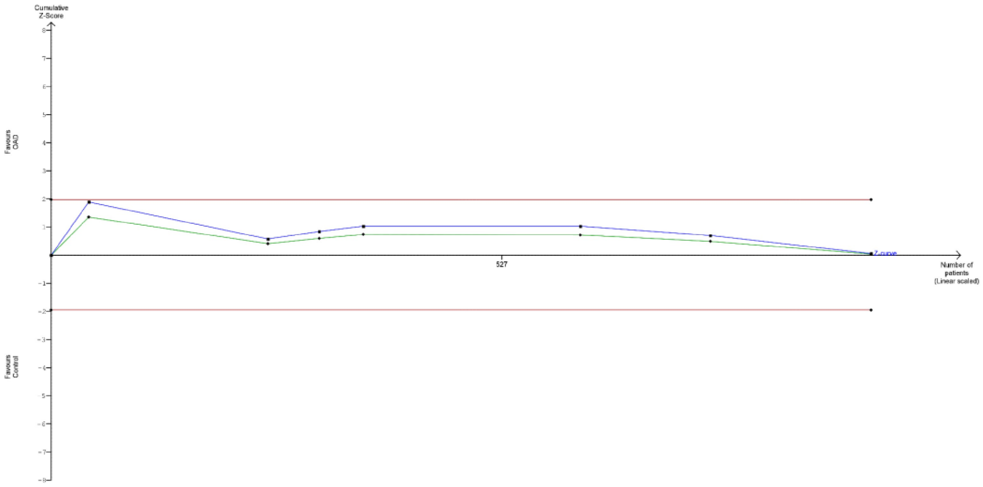

(Supplementary fig.5) TSA sensitivity analysis on MDS-UPDRS I (on medication)

MDS-UPDRS I penalized Z-curve not passing the conventional boundary

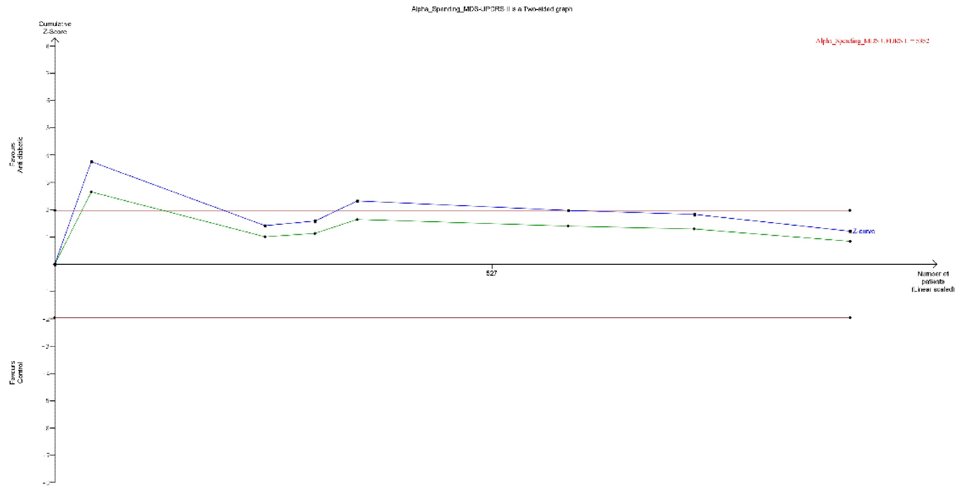

(Supplementary fig.6) TSA sensitivity analysis on MDS-UPDRS II (on medication)

MDS-UPDRS II penalized Z-curve not passing the conventional boundary

MDS-UPDRS III on-medication

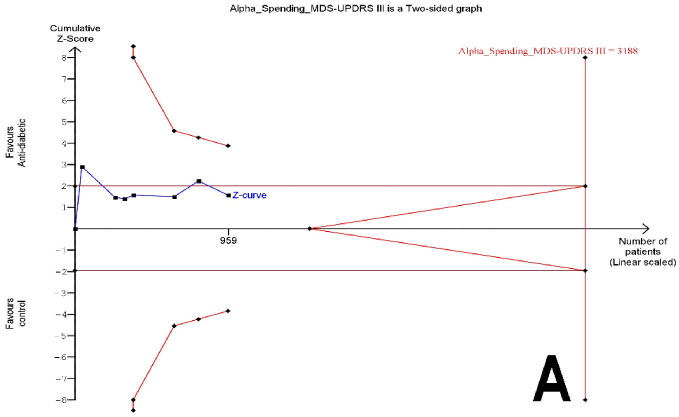

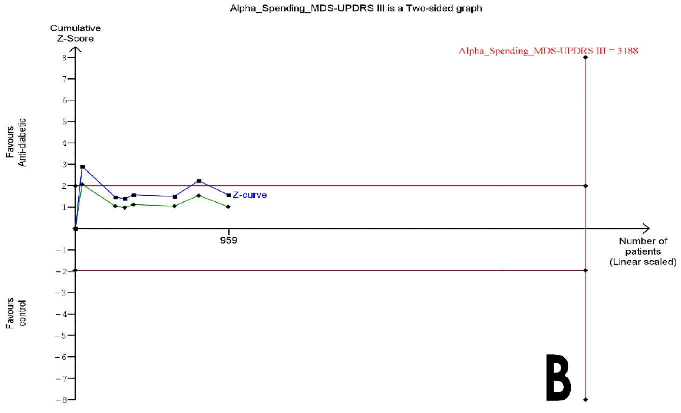

(Supplementary fig.7) TSA sensitivity analysis on MDS-UPDRS III (on medication)

a) MDS-UPDRS III cumulative z-curve not passing the superiority boundary (False positive), b) MDS-UPDRS III penalized Z-curve not passing the conventional boundary

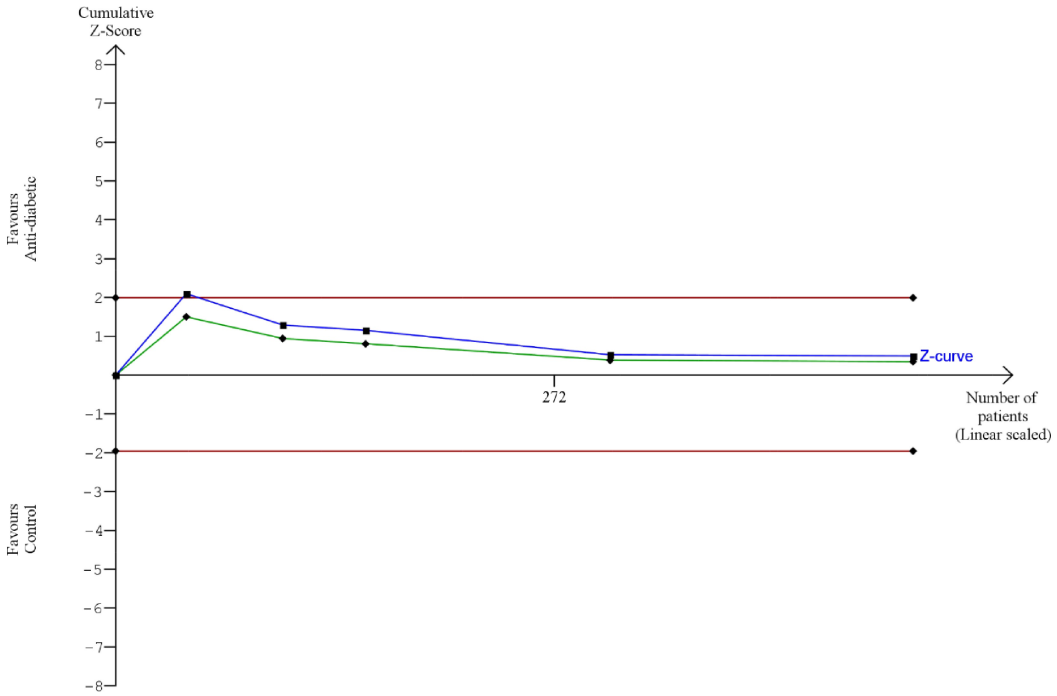

(Supplementary fig.8) TSA sensitivity analysis on MDS-UPDRS IV (on medication)

MDS-UPDRS IV penalized Z-curve not passing the conventional boundary

MDS-UPDRS III off-medication

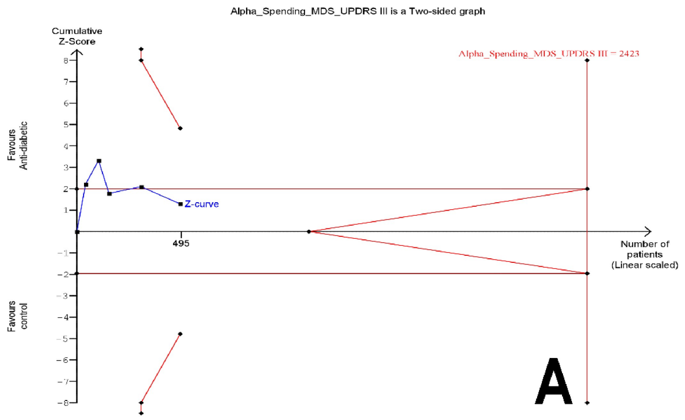

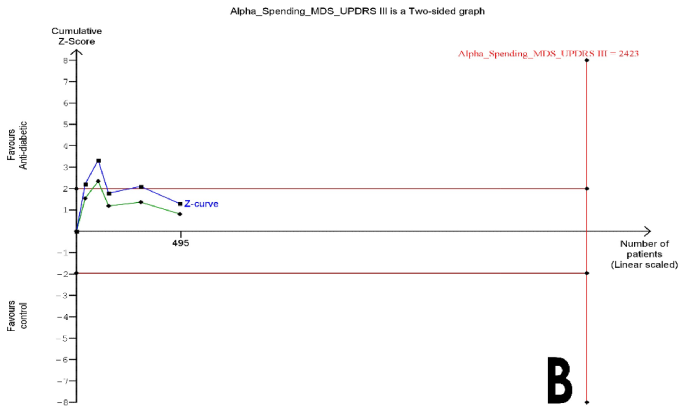

(Supplementary fig.9) TSA sensitivity analysis on MDS-UPDRS III (off medication)

a) MDS-UPDRS III cumulative z-curve not passing the superiority boundary (False positive), b) MDS-UPDRS III penalized Z-curve not passing the conventional boundary

(Table S1) Details of search strategy among databases

Database	Keywords
PubMed	("Parkinson Disease"[Mesh] OR “Parkinson” OR “Parkinson's” OR "Parkinsonism" OR "Parkinsons" OR "PD") AND ("Hypoglycemic Agents"[Mesh] OR "Antidiabetic" OR "Anti-diabetic" OR "Diabetes" OR "DM" OR "Metformin" OR "Sulfonylureas" OR "Glitazones" OR "Dipeptidyl Peptidase-4 Inhibitors" OR "DPP-4" OR "DDP4" OR "Glucagon-Like Peptide-1" OR "Glucagon-Like Peptide1" OR "Glucagon-Like Peptide 1" OR "GLP-1" OR "GLP1" OR "GLP 1" OR "Sodium-Glucose Cotransporter-2" OR "Sodium-Glucose Cotransporter2" OR "Sodium-Glucose Cotransporter 2" OR "SGLT2" OR "α-Glucosidase" OR "Glinides" OR "Exenatide" OR "Liraglutide" OR "Dulaglutide" OR "Semaglutide" OR "Lixisenatide" OR "Albiglutide" OR "Efpeglenatide" OR "Pioglitazone" OR "Rosiglitazone" OR "NLY01")
Scopus	
WOS	
Cochrane library	
